# Plasmonic ELISA for Biomarker Detection: A Review of Mechanisms, Functionalization Strategies, and Emerging Modalities

**DOI:** 10.1021/acsabm.5c00738

**Published:** 2025-06-17

**Authors:** Chaudhary Ammar Shoukat, Maryam Tariq, Raja Muhammad Aqib, Muhammad Ali Tajwar, Rashid Iqbal

**Affiliations:** † Institute for Advanced Study, 620539 Shenzhen University, Shenzhen 518060, P. R. China; ‡ College of Resources and Environment, University of Chinese Academy of Sciences, Beijing 100049, China; § Department of Mechanical Synthesis Engineering, 26714 Jeonbuk National University, 567 Baekjedaero, Deokji-gu, Jeonju-si, Jeollabuk-do 54896, Republic of Korea; ∥ Beijing National Laboratory of Molecular Sciences, Key Laboratory of Analytical Chemistry for Living Biosystems, Institute of Chemistry, 53030 Chinese Academy of Sciences, Beijing 100190, P. R. China; ⊥ Department of Chemistry, 25809 The University of Hong Kong, Hong Kong 999077, China; # Materials Innovation Institute for Life Sciences and Energy (MILES), HKU-SIRI, Shenzhen 518000, China

**Keywords:** plasmonic ELISA, nanoparticle surface functionalization, biomarker detection, multiplexed biosensing, clinical diagnostics

## Abstract

Plasmonic enzyme-linked immunosorbent assay (ELISA) effectively integrates noble metal nanostructures with traditional immunoassays, facilitating rapid, ultrasensitive, and multiplexed biomarker detection. By leveraging localized surface plasmon resonance modulations instigated by biocatalytic reactions and analyte binding, these assays achieve signal amplification through growth, etching, and aggregation mechanisms. Such methodologies significantly enhance detection limits by factors ranging from 10- to over 1000-fold, attaining sensitivity at the subpicogram per milliliter level. Robust surface functionalization methods, including electrostatic adsorption, covalent coupling, and affinity binding, ensure stable immobilization of antibodies while preserving the activity of the nanozymes. Incorporating advanced two-dimensional nanomaterials, such as graphene derivatives and MXenes, further augments the sensitivity (up to ∼200-fold), assay stability, and potential for miniaturization. Emerging modalities, including electrochemical techniques, microfluidics, photothermal methods, surface-enhanced infrared absorption (SEIRA), surface-enhanced Raman scattering, and CRISPR-enabled ELISA, extend the analytical versatility, multiplexing capabilities, and operational speed. Clinical trials, alongside real-world studies, substantiate the efficacy of plasmonic ELISA platforms in early cancer detection, diagnostic evaluation of infectious diseases, and monitoring cardiovascular biomarkers, demonstrating performance comparable to or exceeding that of traditional methodologies. Despite significant advancements, challenges persist with regard to assay standardization, multiplex integration, and large-scale manufacturing. This review presents a comprehensive overview of recent developments, identifies critical knowledge gaps, and outlines future perspectives to expedite the clinical translation of plasmonic ELISA technologies for precision medicine and global health applications.

## Graphical abstract

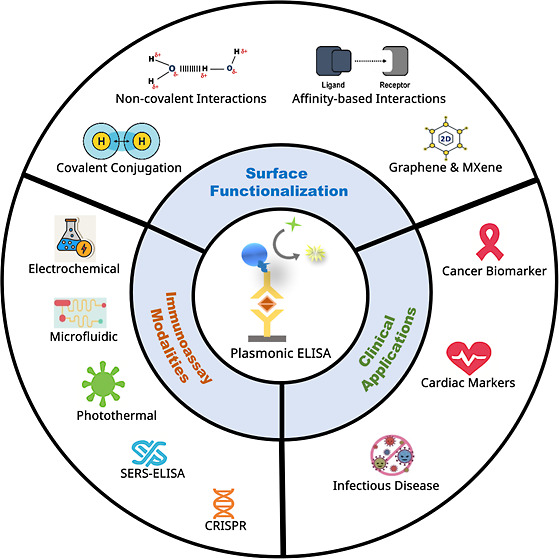

## 1 Introduction

The enzyme-linked immunosorbent assay (ELISA) was first introduced in 1971 by Engvall and Perlman in Sweden, and independently by Schuurs and van Weemen in The Netherlands, as a plate-based technique for detecting and quantifying antibodies, peptides, proteins, and hormones in complex biological samples.^1^ ELISA involves immobilizing the target analyte, such as an antigen, on a microplate, followed by binding with an enzyme-labeled antibody, and subsequent signal amplification through enzymatic reactions. It has become the gold standard for quantifying numerous biomolecules, including disease markers, infectious agents, allergens, and environmental pollutants.^1,2^ Despite its widespread use and well-established protocols, conventional ELISA faces several limitations that restrict its sensitivity, throughput, and applicability. A significant drawback is the fluctuation in detection limits that largely depend on antibody–antigen binding affinities, which can vary considerably across different immunoassay systems.^3^ The extent of signal amplification achievable constrains the detection range through enzyme–substrate reactions.^4^ The necessity for bulky, centralized laboratory equipment such as microplate readers further restricts its portability and point-of-care applications. Additionally, conventional ELISA often necessitates relatively large sample volumes, impeding its use in minimally invasive diagnostics.^1–5^

Integrating plasmonic nanomaterials into ELISA platforms has emerged as a powerful strategy to overcome these challenges, substantially enhancing assay sensitivity, specificity, and multiplexing capability.^6^ Plasmons, collective oscillations of free electrons at metal–dielectric interfaces excited by light, manifest as surface plasmon resonance (SPR) or localized SPR (LSPR) in metallic nanostructures.^7^ The excitation of LSPR generates intense local electromagnetic fields that amplify spectroscopic signals and enable sensitive detection modalities.^8,9^

Figure 1 illustrates the fundamental differences between conventional ELISA and various plasmonic-integrated ELISA platforms, emphasizing key modalities that utilize plasmonic nanomaterials to improve the assay sensitivity and versatility.

**Figure 1:**
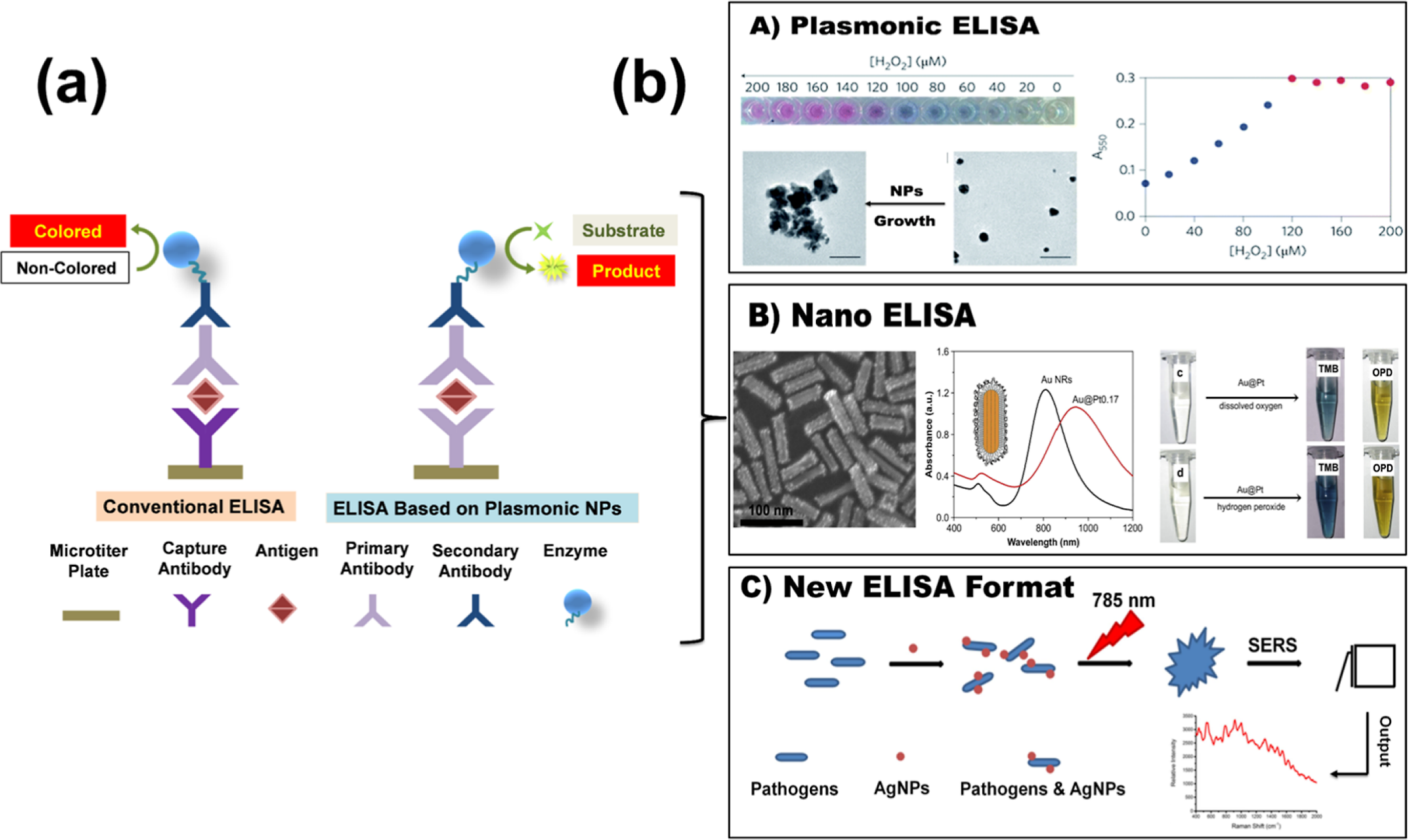
(a) Comparison of conventional ELISA and plasmonic-integrated ELISA, emphasizing enhanced sensitivity and multiplexing via plasmonic nanomaterials through LSPR and nanozyme catalysis. (b) Plasmonic ELISA modalities: (A) plasmonic ELISA achieving ultrasensitive colorimetric detection via noble metal nanoparticles. Reproduced with permission.^10^ Copyright 2016, Royal Society of Chemistry. (B) Nanozyme-linked immunosorbent assay (NLISA) using enzyme-mimicking nanomaterials for signal amplification. Reproduced with permission.^11^ Copyright 2011, Elsevier. (C) Surface-enhanced Raman scattering (SERS)-based ELISA offering highly specific biomarker detection with spectral resolution. Reproduced with permission.^12^ Copyright 2018, Frontiers.

As shown in Figure 1, plasmonic ELISAs utilize noble metal nanostructures’ unique optical and catalytic properties to achieve enhanced detection performance. This enhancement underpins several field-enhanced spectroscopic techniques, including SERS,^13^ metal-enhanced fluorescence (MEF),^14^ and surface-enhanced infrared absorption (SEIRA).^15^ Furthermore, the intense light absorption associated with plasmons produces significant photothermal effects that can accelerate enzymatic reactions or can be exploited for photothermal imaging. Plasmonic photocatalysis leverages both electromagnetic field enhancement and the generation of energetic hot carriers to catalyze chemical reactions, further expanding assay functionality.^16,17^ The sensitivity and specificity of plasmonic ELISA depend critically on nanoparticle properties such as size, shape, and composition. For instance, tuning gold nanorods, nanospheres, or core–shell nanoparticles modifies their LSPR peak positions and scattering behaviors, optimizing interactions with target biomolecules for enhanced detection accuracy.^18,19^ Plasmonic photocatalysis is another benefit of local plasmons, which can utilize field enhancement and hot carriers.^16^ Different metallic nanostructures have been demonstrated for developing ELISA platforms, including solid NPs,^20^ nanoholes,^21^ nanorods,^22^ fractals,^23^ branched structures,^24^ nanowires,^25^ quantum dots,^26^ hybrid nanostructures,^27^ nanopyramids,^28^ metal organic frameworks,^29^ and core–shell nanoparticles.^30^ Plasmonic nanostructures can also substitute traditional organic chromogenic substrates (e.g., TMB and OPD), producing vivid color changes that are readily visible to the naked eye, thus facilitating rapid point-of-care diagnostics without requiring specialized instrumentation.^31^ Recent advances have incorporated novel 2D nanomaterials such as graphene oxide (GO), reduced graphene oxide (rGO), and MXenes into plasmonic ELISA platforms. These offer improved biocompatibility, enhanced electron transfer, and increased antibody loading capacity.^32,33^ MXene composites exhibit accordion-like layered structures with abundant surface functional groups, preventing nanoparticle aggregation and boosting signal amplification for ultrasensitive detection of cancer biomarkers such as carcinoembryonic antigen (CEA).^34^ These characteristics translate into improved detection limits and expanded dynamic ranges in clinical assays. Beyond traditional approaches, emerging modalities have extended plasmonic ELISA’s capabilities, including integration with CRISPR/Cas systems for nucleic acid recognition, microfluidic devices for multiplexed assays, and wearable biosensor platforms for real-time physiological monitoring.^35–37^ CRISPR-Cas12a-assisted dual-mode aptasensors combine fluorescent and lateral flow readouts to enable rapid, highly specific detection of environmental toxins and infectious agents on-site.^38^ Wearable nanozyme-enzyme electrochemical biosensors have been demonstrated for continuous lactate monitoring in sweat, underscoring the potential for personalized health management.^39^ Plasmonic ELISA has exhibited clinical utility across multiple fields. Cancer biomarkers such as prostate-specific antigen (PSA), carcinoembryonic antigen (CEA), and glial fibrillary acidic protein (GFAP) have been detected with enhanced sensitivity using plasmonic nanomaterials.^40–42^ Infectious disease markers, including the SARS-CoV-2 spike protein and carbohydrate antigen 19–9 (CA19–9), also benefit from these advancements, facilitating earlier and more accurate diagnoses.^43,44^ Notably, cardiac biomarkers such as cardiac troponin I (cTnI) have been effectively quantified with improved sensitivity and specificity, facilitating timely diagnoses of acute myocardial infarction and enhancing patient outcomes.^45^ Therefore, integrating plasmonic nanotechnology with ELISA methodologies has revolutionized biomarker detection by offering increased sensitivity, expanded multiplexing capabilities, and adaptability to advanced diagnostic technologies.^46,47^ Plasmon-integrated ELISAs can enhance sensitivity through improved light absorption via various regulation modes, augmented catalytic activity through the introduction of nanozymes, and more sensitive signal transduction mechanisms associated with local plasmons.^48^ For instance, by controlling the orientation of capture antibodies on AuNPs, the sensitivity of ELISA-based medical devices is significantly enhanced.^49^ The large surface area of plasmonic nanoparticles facilitates multilabeling capabilities. Multiple antibodies can be conjugated to a single nanoparticle (for example, at different spatial sites with distinct fluorescent molecules) to achieve multiplex detection.^46^ A slight alteration in the refractive index of the surrounding medium can result in a peak shift of LSPR bands, thereby enabling the detection of target antigen binding.^8^ The geometric configuration of the nanostructures may also influence the SPR responses, leading to unique scattering patterns and allowing for more precise detection of the target antigen.^47^ The assay’s sensitivity and accuracy are enhanced by effectively encapsulating antibodies or antigens and amplifying fluorescence signals.^50^ The utilization of plasmonic nanostructures to minimize background noise improves detection accuracy and precision, facilitating the identification of lower analyte concentrations and specific analytes.^51^ Enhanced signal amplification, including improved light absorption, photoluminescence, and electrochemiluminescence, can bolster assay response and measurement accuracy while reducing assay duration.^52^ Furthermore, hybrid plasmonic systems can yield superior optical properties when combined with other materials. These innovations hold significant potential for advancing the development of portable, rapid, and reliable diagnostic tools that are critical for clinical and field applications.

## 2 Optical Properties of Plasmonic Nanomaterials

Localized plasmons surpass the limitations of conventional detection methods by creating strong electromagnetic fields at metallic nanostructures.^7^ This enhancement of optical signals enables the implementation of advanced techniques, including SERS,^13^ MEF,^50^ and SEIRA.^15^ By tuning the properties of nanoparticles, plasmonic materials enhance sensitivity and enable innovative biosensing methods, which include photothermal and photocatalytic applications.^53^

### 2.1 Localized Surface Plasmon Resonance: Impact of Nanoparticle Design on Sensitivity and Specificity

The exceptional sensitivity exhibited by plasmonic ELISA can primarily be attributed to the capability of metallic nanoparticles to concentrate electromagnetic fields at their surfaces through the mechanism of LSPR^7,54^ (Figure 2A). By meticulously engineering parameters such as nanoparticle size, shape, material composition, and morphology, researchers have accomplished enhancements in the limit of detection (LOD) of up to 3 orders of magnitude, all while concurrently maintaining and, in certain cases, improving the assay’s specificity.^55^ Table 1 delineates key representative studies that exemplify the correlation between these design parameters and the observable improvements in sensitivity and specificity across various assay formats.

**Figure 2:**
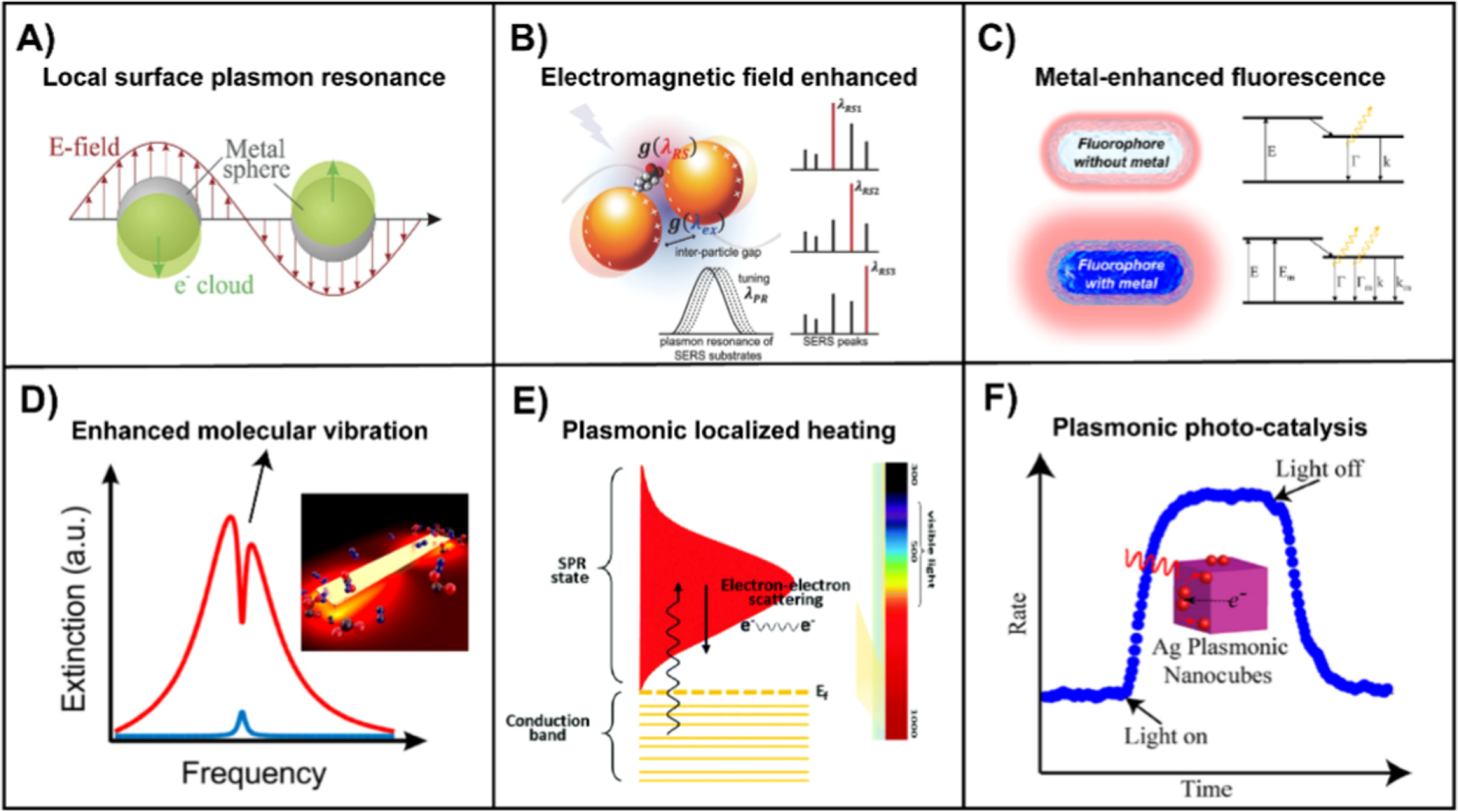
Unique properties of plasmonic nanostructures: (A) localized SPR. Reproduced with permission.^54^ Copyright 2022, MDPI. (B) SERS via local EF enhancement. Reproduced with permission.^78^ Copyright 2014, Wiley. (C) MEF via increased radiative rate (Γm) in the presence of nearby AuNR in comparison to that (Γ) in the absence of the AuNR. Reproduced with permission.^74^ Copyright 2023, American Chemical Society. (D) SEIRA. Reproduced with permission.^76^ Copyright 2017, American Chemical Society. (E) Photothermal effect. Reproduced with permission.^79^ Copyright 2019, Royal Society of Chemistry. (F) Plasmon-enhanced photocatalysis. Reproduced with permission.^80^ Copyright 2013, American Chemical Society.

**Table 1:** Effects of Nanoparticle Design and Morphology on Plasmonic ELISA Performance

nanostructure type	variation	limit of detection (LOD)	comparison with traditional ELISA	ref.
size (Gold nanoparticles)	12.27 nm	21.87 fg/mL for FL and 17.06 fg/mL for SERS	faster detection, lower interference, improved accuracy for OTA detection	^56^
	15 nm Au spheres	0.275 ng/mL	good accuracy and selectivity for OTA detection	^57^
	20 nm Au spheres	11 pg/mL	faster detection and reduced interference for more accurate and reliable assays for OTA detection	^58^
shape (Gold)	nanorods	86 pg/mL	rapid and affordable detection for anti-Müllerian hormone (AMH)	^59^
	nanostars	38 ng/mL	improved reproducibility and sensitivity for human IgG detection	^13^
	nanoprism	100 fM for human α-thrombin and 1 pM VEGF-165	low cost, high specificity, and stability with remarkable sensitivity	^60^
material	silver nanoparticles	8 pg/mL	Superior sensitivity, wider dynamic range, and enhanced specificity for Parkinson’s disease biomarkers	^61^
	rhodium nanocatalyst	1.2 pg/mL	250-fold increased sensitivity, rapid, low-cost, and high stability for the detection of staphylococcal enterotoxin B	^62^
	platinum nanoparticles	38.7 pg/mL	Higher sensitivity, wider range, and enzyme-free for α-fetoprotein detection	^63^
nanoparticle morphologies	Fractals (gold)	88 PFU/mL	label-free, higher sensitivity, rapid detection for SARS-CoV-2 Detection	^64^
	branched nanostructures (SiO_2_@AuPt)	0.33 pg/mL	high sensitivity, improved feasibility for the detection of squamous cell carcinoma antigen	^65^
	nanowires (silicon nanowire arrays)	1.47 pg/mL	stable and reproducible, high sensitivity and selectivity for efficient detection of myocardial infarction	^66^
	hybrid nanostructures (Fe_3_O_4_ magnetic nanoparticles in fluorescent polymer dots)	2.15 ng/mL	fast and low-cost detection for the detection of two Mycotoxins	^67^
	nanopyramids (gold nanopyramid arrays)	0.6 pg/mL	improved detection sensitivity for the detection of traumatic brain injury biomarker	^68^

The design of nanoparticles significantly influences the performance of ELISA, particularly in terms of detection sensitivity and assay reliability. The size of AuNPs is a critical determinant; for example, 15 nm Au spheres achieve a LOD of approximately 0.275 ng/mL, demonstrating commendable selectivity for ochratoxin A (OTA).^57^ Increasing the size to approximately 20 nm spheres enhances detection capabilities to 11 pg/mL, facilitating faster and more accurate OTA assays while concurrently reducing interference.^58^ Even smaller AuNPs, such as 12.27 nm, when combined with fluorescence (FL) or SERS, attain ultralow LODs in the fg/mL range for OTA, significantly outperforming traditional ELISAs.^56^

Furthermore, the shape of nanoparticles increases sensitivity through the formation of plasmonic “hot spots”. Gold nanorods exhibit rapid and cost-effective detection abilities, achieving LODs near 86 pg/mL for anti-Mullerian hormone (AMH).^59^ Nanostars offer enhanced reproducibility and sensitivity, with detection limits of 38 ng/mL for human immunoglobulin G (IgG).^13^ At the same time, nanoprism structures achieve ultrahigh specificity and stability with detection limits as low as 100 fM for human α-thrombin and 1 pM for VEGF-165.^60^

Moreover, the material composition is pivotal in enhancing the assay performance. Silver nanoparticles attain LOD as low as 8.0 pg/mL, offering superior sensitivity and specificity for biomarkers related to Parkinson’s disease.^61^ Other materials, such as rhodium and platinum nanoparticles, have exhibited significant enhancements in sensitivity, with LODs of 1.2 pg/mL and 38.7 pg/mL, respectively, in relevant bioassays.^62,63^

Advanced morphologies and hybrid nanostructures contribute to increased versatility and performance of the assays. Gold fractals enable label-free rapid detection of SARS-CoV-2 with SiO_2_ approaching 88 PFU/mL.^64^ Branched SiO_2_@Aupt nanostructures achieve high sensitivity (0.33 pg/mL) for squamous cell carcinoma antigen (SCCA).^65^ Silicon nanowire arrays provide stable and selective detection of myocardial infarction markers with LODs of approximately 1.47 pg/mL.^66^ Hybrid Fe_3_O_4_ magnetic nanoparticles embedded in fluorescent polymer dots facilitate the rapid and cost-effective detection of mycotoxins at approximately 2.15 ng/mL.^67^

Overall, optimizing the size, shape, material, and morphology of plasmonic nanoparticles can reduce detection limits from nanogram to femtogram levels, enhance assay speed and accuracy, and enable multiplexed clinical diagnostics and environmental monitoring with improved specificity and reproducibility.

### 2.2 Surface-Enhanced Raman Scattering

SERS represents a highly promising technique for sensing applications.^13,28^ When molecules are adsorbed in proximity to the surfaces of metallic nanoparticles, such as silver or gold nanoparticles, their Raman signals exhibit substantial enhancement attributable to electromagnetic field (EF) enhancement and chemical enhancement (Figure 2B).^69^ By optimization of the local EF enhancement through the creation of hot spots, the intensities of the Raman signals can be significantly increased, which is advantageous for detecting molecules at low concentrations. Under optimized conditions, enhancements of up to 10̂14 orders of magnitude allow for the detection of individual molecules.^70,71^

### 2.3 Metal-Enhanced Fluorescence (MEF)

MEF, commonly called plasmon-enhanced fluorescence, significantly enhances weak fluorophores’ fluorescence quantum yield through local field enhancement facilitated by local plasmons.^14^ In contrast to surface-enhanced Raman Spectroscopy (SERS), it is imperative to maintain an appropriate distance between the fluorophore and the metal surface, as excessive proximity may result in the quenching of fluorescence via two energy transfer mechanisms: metal surface energy transfer and Förster resonance energy transfer. This distance must exceed 5 nm, typically from 5 to 90 nm.^72,73^ Beyond the enhancement in emission brightness, improved photostability is also observed due to fluorophore immobilization. The fluorescence enhancement of Cy5 utilizing mesoporous silica-coated AuNRs was investigated. With a silica thickness of 10 nm, they achieved an 8-fold enhancement in fluorescence, coupled with a reduced fluorescence lifetime. They attributed this observation to the increased radiative decay rate (*m*) of Cy5 in the presence of AuNRs, as depicted in Figure 2C, which contributes to an enhancement of the fluorescence quantum yield and a reduction in the fluorescence lifetime. By employing this enhanced fluorescence probe, they successfully facilitated the highly sensitive detection of the influenza A virus using a lateral flow immunosensor, achieving an LOD of 0.52 pg mL^–1^ within a time frame of 20 min. It is evident that the MEF effect can be utilized to augment the sensitivity of fluorescence-based detection modalities.^74^

### 2.4 Surface-Enhanced Infrared Absorption

Similar to Surface-Enhanced Raman Spectroscopy (SERS), the enhancement of the local electric field may be utilized to amplify the vibrational absorption of molecules, a phenomenon known as surface-enhanced infrared absorption (SEIRA).^15,75^ In particular, when the plasmon, represented in red, aligns with the molecular vibration, represented in blue, the infrared vibration of the molecules located within the enhanced electromagnetic near-field of the plasmonic nanostructure, or nanoantenna, experiences significant intensification (Figure 2D).^76,77^

### 2.5 Photothermal Effect

The excitation of local plasmons not only induces a significant near-field enhancement effect at the surface of plasmonic nanoparticles but also generates localized heating due to the nonradiative decay of high-energy electrons (Figure 2E).^79,81^ This phenomenon leads to a pronounced photothermal effect, which is attributed to a substantial absorption cross section, further enhanced by the high regulation capability of localized plasmons. This distinctive aspect of localized heating is the subject of extensive research for tumor treatment and biological imaging, achieved by adjusting the responses of LSPR (SPR) within the near-infrared I and II regions.^17^

### 2.6 Plasmonic Photocatalysis

Plasmon nanomaterials represent a novel class of photocatalytic materials. On one hand, they offer enhanced reactant excitation through localized electric field (EF) enhancement. On the other hand, the generated hot carriers can directly participate in photochemical reactions.^16,82^ As illustrated in Figure 2F, when Ag nanocubes are used as the photocatalyst, local plasmon excitation results in approximately a 4-fold increase in the steady-state rate of ethylene epoxidation compared to the thermal process.^80^ Plasmonic photocatalysis provides a way to modulate the nanozyme activity using light. The local plasmon-enhanced properties can be integrated into conventional ELISA to address their limitations and improve detection modalities.^83,84^

## 3 Plasmonic Enzyme-Linked Immunosorbent Assay (p-ELISA)

Plasmonic Enzyme-Linked Immunosorbent Assay (p-ELISA) represents an innovative modification of the conventional ELISA, incorporating its specific recognition advantages alongside the high extinction cross sections of plasmonic nanomaterials utilized as chromogenic substrates.^6,20,37^ In conventional ELISA, signal amplification is achieved through an enzyme-mediated redox reaction, which results in alterations in absorption or fluorescence.^1,2^ Conversely, in p-ELISA, color development is predicated upon modifications of the LSPR (SPR) properties, induced by the enzyme-mediated redox reaction.^6,20,37^ p-ELISA significantly enhances the sensitivity and performance of bioassays. Given that plasmonic nanoparticles are employed solely as chromogenic substrates, p-ELISA demonstrates optimal compatibility with conventional ELISA compared with various plasmon-integrated ELISAs. Notably, in the concluding phase, plasmonic NPs are introduced to facilitate color development through the products of enzyme-mediated reactions, which subsequently trigger particle growth, etching, or aggregation.^40^

### 3.1 Growth-Based p-ELISA Methodology

In the context of growth-based p-ELISA, a growth solution is introduced in the presence of NP seeds. The product of an enzyme-mediated reaction, such as hydrogen peroxide, facilitates the deposition of metal ions (such as Au^3+^ or Ag^+^) onto the seeds.^85–87^ The growth-induced alterations in the localized SPR band yield a colorimetric signal that can be perceived with the naked eye or quantified using a microplate reader.^10,85,86^ For instance, Wang et al. illustrated a visual detection method for HER2 ECD, a breast cancer biomarker pertinent to the early diagnosis of HER2-dependent breast cancers, through ascorbic acid (AA)-mediated growth of Au nanobipyramids (AuNBPs).^85^ As exhibited in Figure 3, with an increase in the HER2 ECD concentration, additional AA molecules were released from l-ascorbic acid 2-phosphate salt (AAP) via hydrolysis induced by alkaline phosphatase (ALP). The resultant AA reduced Au­(I) ions to the seeds, which subsequently led to the formation and growth of AuNBPs. The alteration in the aspect ratio of the AuNBPs resulted in a conspicuous color change, thereby enabling multicolor visual detection of HER2 ECD. The minimum detectable concentration is 0.5 ng/mL, as determined by naked eye observation.

**Figure 3:**
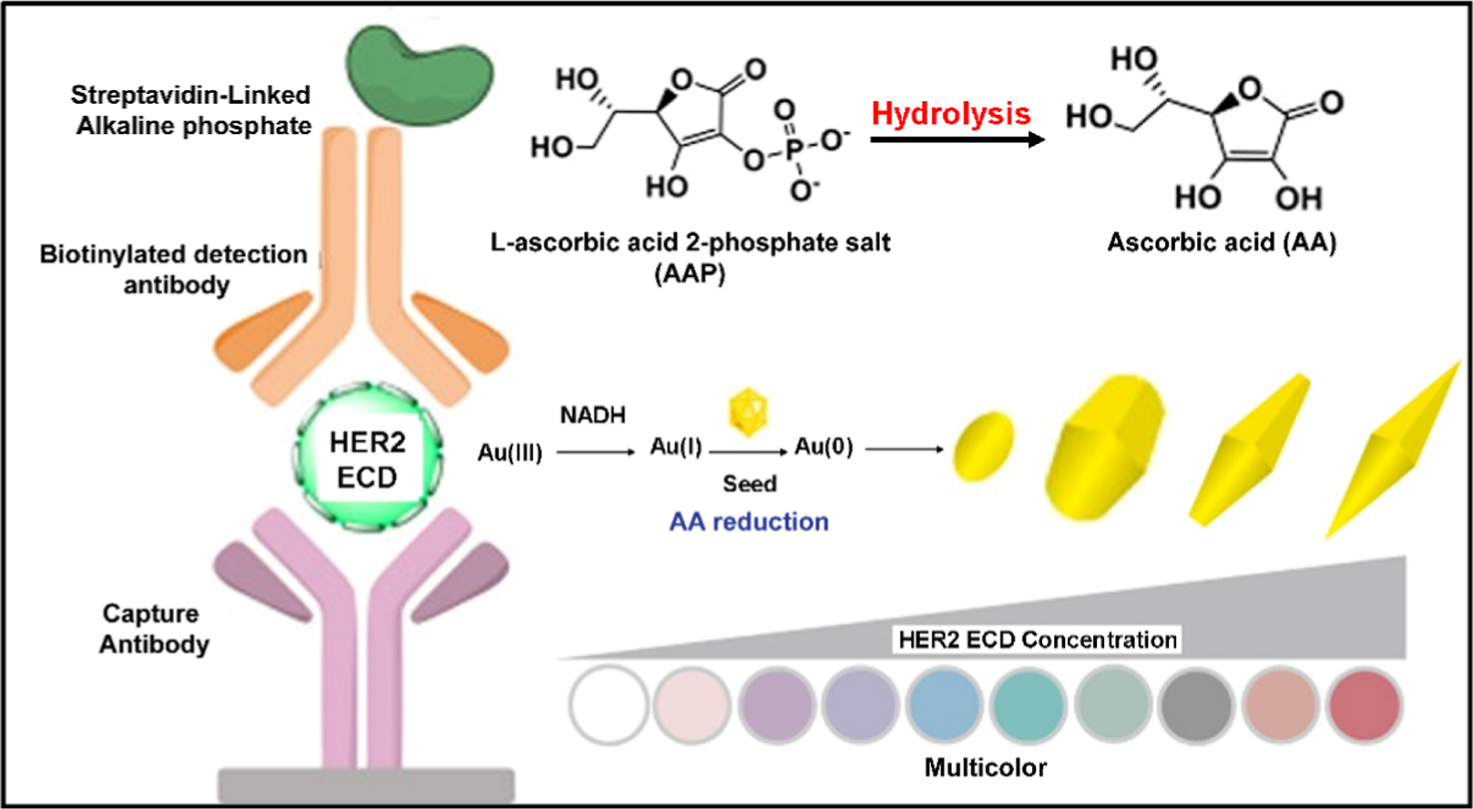
Illustration of the growth-based Plasmonic-ELISA for multicolor visual detection of HER2 ECD. The assay employs NADH-assisted and AA-mediated growth of gold nanobipyramids (Au NBPs). The subsequent size-dependent optical shift facilitates colorimetric detection that corresponds to the HER2 ECD concentration through antibody recognition. Reproduced with permission.^85^ Copyright 2020, American Chemical Society.

A p-ELISA for detecting alpha-fetoprotein (AFP) was demonstrated, utilizing ALP-mediated AgNP growth, achieving an LOD as low as 0.23 ng/mL, observable with the naked eye. This sensitivity was approximately ten times greater than a conventional HRP-based ELISA.^88^ Similarly, leveraging the ALP-mediated growth of AgNPs, Shaban et al. presented a sensing method for ALP, which exhibited a dynamic linear range of 0.5 to 225 U/L with a detection limit of 0.24 U/L.^86^

### 3.2 Etching-Based p-ELISA

In contrast to the growth-based p-ELISA, the etching-based p-ELISA utilizes the product of the enzyme-mediated reaction as an etchant to etch large plasmonic nanoparticles.^89^ The etching process modifies the plasmonic responses, which serve as the readout.^89,90^ Jiang et al. present a novel, simple, and sensitive visual detection method for the heart failure biomarker NT-proBNP by integrating traditional double-antibody sandwich ELISA with a plasmonic signal readout based on AuNR etching. In this methodology, the presence of NT-proBNP is captured between two antibodies, with the detection antibody labeled with horseradish peroxidase (HRP). Upon the addition of the substrate TMB, HRP catalyzes its oxidation to TMB^2+^, which functions as an etching agent that selectively reduces the length of AuNRs. This etching results in a measurable blue shift in the longitudinal LSPR peak of the AuNRs, culminating in a visible color change from brown to blue and eventually yellow, which directly correlates with the NT-proBNP concentration. Figure 4 schematically illustrates this process, demonstrating how antigen–antibody binding leads to HRP-mediated generation of TMB^2+^, which etches AuNRs and produces the plasmonic signal, enabling naked-eye detection without the need for complex instruments and providing a practical approach for point-of-care diagnostics.^91^

**Figure 4:**
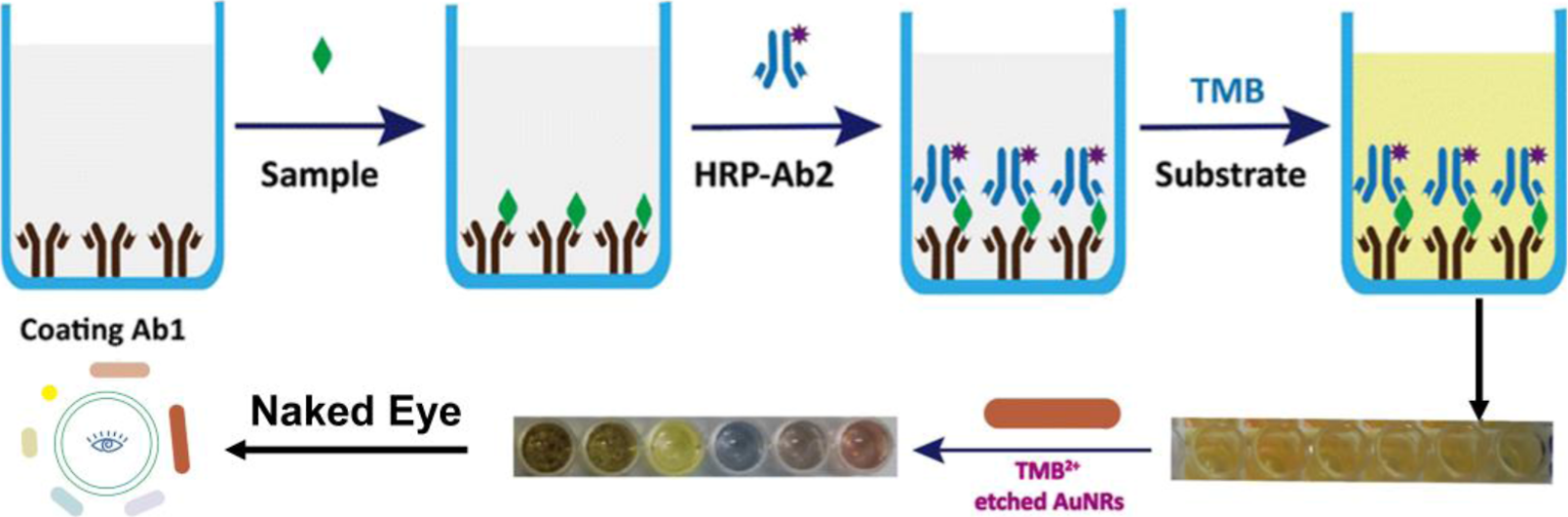
Illustration of the etching-based plasmonic enzyme-linked immunosorbent assay (ELISA) for the detection of NT-proBNP utilizing TMB^2+^-mediated alteration of the shape of gold nanorods. The immunoassay initiates the HRP-driven oxidation of TMB, resulting in the production of TMB^2+^, which etches the AuNRs and induces a visible color change that is proportional to the concentration of the biomarker. Reproduced with permission.^91^ Copyright 2023, Royal Society of Chemistry.

Ma et al. presented an etching-based plasmonic ELISA to detect trace silver ions (Ag­(I)) via Ag­(I)-inhibited platinum nanozymes that etch gold nanorods. This process causes a blue shift and color change proportional to the Ag­(I) concentration, with a detection limit as low as 20 nm. The assay utilizes platinum nanoparticles (PtNPs) whose catalase-like activity is inhibited by Ag­(I) binding, thereby reducing hydrogen peroxide decomposition and generating hydroxyl radicals (OH) for etching AuNRs. This decreases their aspect ratio and shifts their LSPR spectrum, producing color changes proportional to the Ag­(I) levels. In contrast to conventional methods, which exhibit inverse analyte correlations, this platform provides direct, sensitive, and visible multicolor responses for low-concentration detection, making it promising for environmental and food safety monitoring.^92^

### 3.3 Aggregation-Based p-ELISA

Aggregation-based-ELISA utilizes the phenomenon of LSPR, exhibited by metallic nanoparticles such as AuNPs and AgNPs, to create a visible color change that serves as the detection signal.^40,93^ In their nonaggregated state, spherical AuNPs and AgNPs suspensions appear as red and yellow, respectively. Upon plasmon coupling, evident in their aggregates, the red shift of LSPR bands triggers a distinct color change, appearing as blue for AuNPs and gray/brown for AgNPs.^10^ This color change resulting from plasmon coupling can be easily observed with the naked eye or quantified using a microplate reader.^10,40,93^ An oxidized low-density lipoprotein (oxLDL) is a biomarker for atherosclerosis and cardiovascular disease. Through salt-induced AuNP aggregation, combined with an oxLDL-selective aptamer, Khongwichit et al. developed a straightforward and rapid assay for detecting oxLDL.^94^ As shown in Figure 5, in the absence of oxLDL, negatively charged aptamers bind to the surface of AuNPs, inhibiting their aggregation under high salt conditions. Conversely, in the presence of oxLDL, the selective binding of aptamers to oxLDL decreases the electrostatic repulsion between AuNPs, leading to their aggregation.

**Figure 5:**
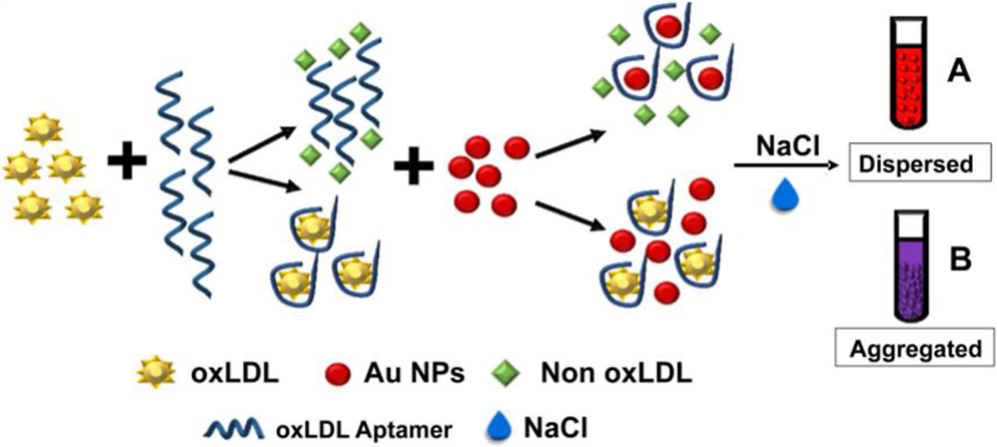
Presentation of an aggregation-based plasmonic enzyme-linked immunosorbent assay (ELISA) designed for the detection of oxLDL utilizing aptamer-functionalized AuNPs. In this procedure, the addition of sodium chloride (NaCl) facilitates aggregation, leading to a noticeable color change from red (dispersed) to purple (aggregated) upon the presence of oxLDL. Reproduced with permission.^94^ Copyright 2023, Elsevier.

The assay exhibited a linear detection range of 0.002–0.5 μmol/L oxLDL, demonstrating a high selectivity for other serum proteins. Similarly, Mahjub et al. developed an AgNP aggregation-based p-ELISA for tobramycin (TOB) detection.^95^ A TOB-specific aptamer was used to coat the AgNPs. In the presence of the cationic polyelectrolyte PDDA (polydiallyldimethylammonium chloride), the formation of the AgNP@aptamer@PDDA complex kept the AgNPs dispersed. Following the introduction of TOB, the dissociation of the complex caused aggregation of AgNPs. Using this detection mechanism, the visual LOD observable to the naked eye was found to be 1 nM. The linear detection range extended from 0.1 to 100 nM, with a quantitative LOD of 70 pM.

## 4 Surface Functionalization of Plasmonic Nanomaterials Binding to Antibodies: Traditional Approaches and Emerging Two-Dimensional (2D) Materials

Surface functionalization plays a pivotal role in designing plasmonic ELISA platforms by facilitating the immobilization of antibodies and antigens on nanoparticle surfaces. The immobilization strategy directly influences the sensitivity, specificity, reproducibility, and overall robustness of the assay.^96–98^ Traditionally, the foundations of biofunctionalization for plasmonic nanomaterials have been established through noncovalent interactions, covalent conjugation, and affinity-based interactions, including the biotin–avidin system.^99^ Nonetheless, the emergence of advanced two-dimensional (2D) materials, particularly derivatives of graphene and MXenes, has unveiled new opportunities for enhancing biosensor performance via improved electronic properties, increased surface areas, and tunable chemistries.^100,101^ This section elaborates on each functionalization method, examining its mechanisms, advantages, and limitations and providing a comparative analysis of their performance within a comprehensive framework.

### 4.1 Non-Covalent Interactions

Noncovalent adsorption represents the most straightforward and widely employed technique for immobilizing antibodies on plasmonic nanomaterials. This process relies on electrostatic forces, hydrophobic interactions, van der Waals forces, and hydrogen bonding to reversibly anchor biomolecules to the surfaces of nanoparticles without the necessity for chemical modification.^102–104^ For instance, hollow platinum nanocage mesoporous silica nanoparticles (HPNMSNs) have noncovalently adsorbed measles antigens, yielding an enhancement in nanozyme catalytic activity and a 3 orders of magnitude improvement in the LOD as compared to conventional HRP-based ELISA, as shown in Figure 6.^105^ This method is regarded as appealing due to its simplicity and gentle conditions, which serve to minimize biomolecule denaturation. Nonetheless, the noncovalent interactions exhibit significant challenges due to poor regulation of protein orientation and density, potentially resulting in diminished bioactivity and increased nonspecific adsorption. Such issues can adversely impact assay reproducibility and specificity, particularly in complex matrices where competitive adsorption may arise. Consequently, while noncovalent strategies prove effective for proof-of-concept studies and rapid prototyping, they necessitate the incorporation of blocking agents and the optimization of surface chemistry to alleviate nonspecific effects.

**Figure 6:**
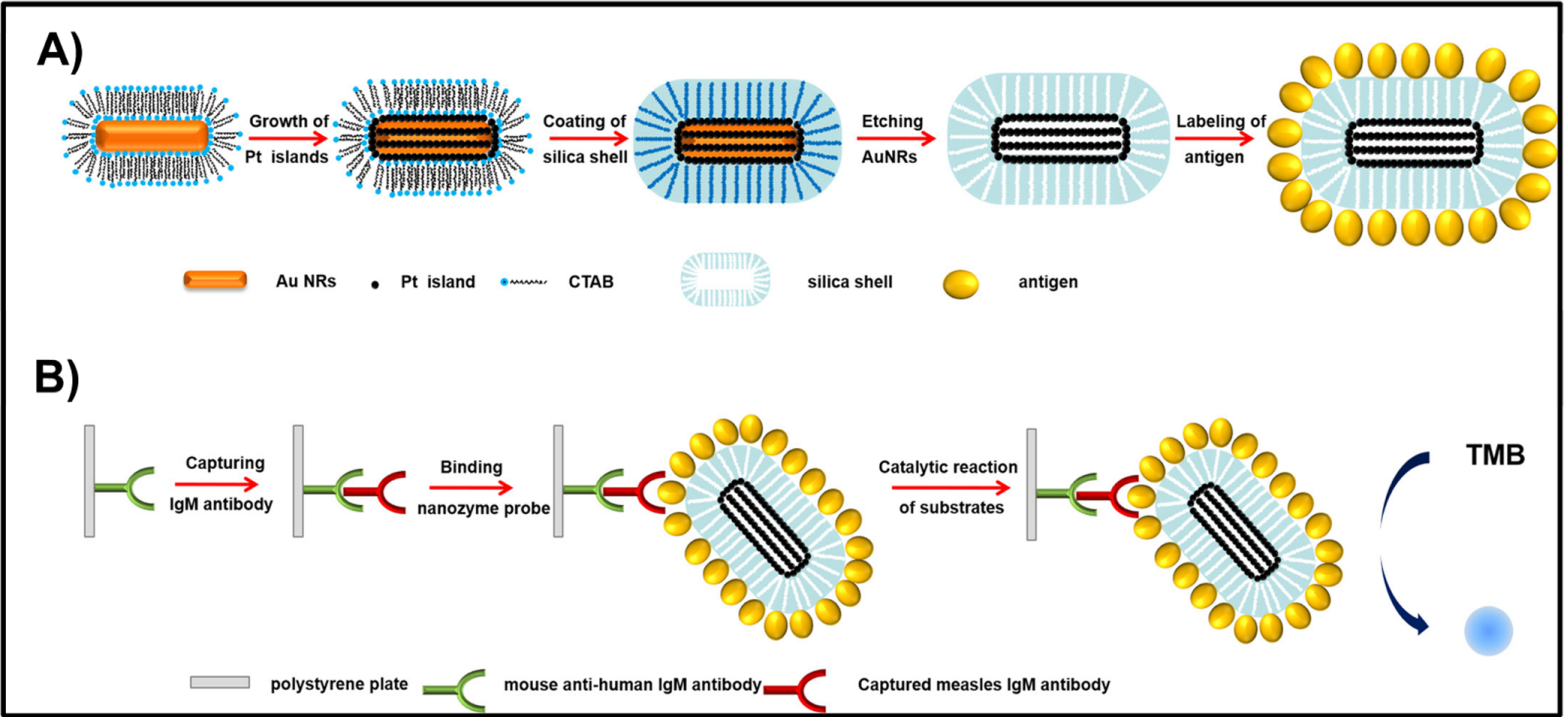
Noncovalent adsorption of antibodies onto HPNMSNs. Antigens adsorb via electrostatic and hydrophobic interactions, preserving the nanozyme catalytic activity and enhancing immunoassay sensitivity. Reproduced with permission.^106^ Copyright 2022, American Chemical Society.

Noncovalent functionalization is both facile and effective in enhancing the sensitivity of ELISA through nanozyme platforms; however, challenges associated with controlling protein orientation and nonspecific binding restrict its robustness. Consequently, additional surface engineering is frequently necessary to optimize the precision of the assay.

### 4.2 Covalent Conjugation

Covalent conjugation promotes stronger, more stable, and often oriented attachment of antibodies to plasmonic nanoparticles, thereby enhancing the reliability and sensitivity of the assays. Standard chemistries used in this context include Au–S bonding on gold surfaces by poly­(ethylene glycol) linker, and 1-ethyl-3-(3-(dimethylamino)­propyl) carbodiimide (EDC) and Sulfo-NHS (*N*-hydroxysulfosuccinimide) reaction coupling, which targets amine and carboxyl groups on proteins and nanoparticle coatings.^106–108^ For instance, mesoporous silica-coated platinum nanozymes, functionalized via EDC/NHS chemistry for the immobilization of hCG antibodies, demonstrated a LOD (c) as low as 10 ng/mL and a broad detection range (Figure 7).^109^ Likewise, Ru nanoparticle-based immunosensors that were covalently functionalized showed a 140-fold increase in sensitivity compared to conventional HRP ELISA for alpha-fetoprotein.^110^ Covalent conjugation reduces antibody desorption, stabilizes orientation, and enhances reproducibility, enabling a clinical-grade assay performance. However, it is crucial to acknowledge that harsh conjugation conditions may lead to protein denaturation and random coupling can adversely affect antigen accessibility. Optimizing conjugation parameters and implementation of site-specific labeling can help mitigate these issues.

**Figure 7:**
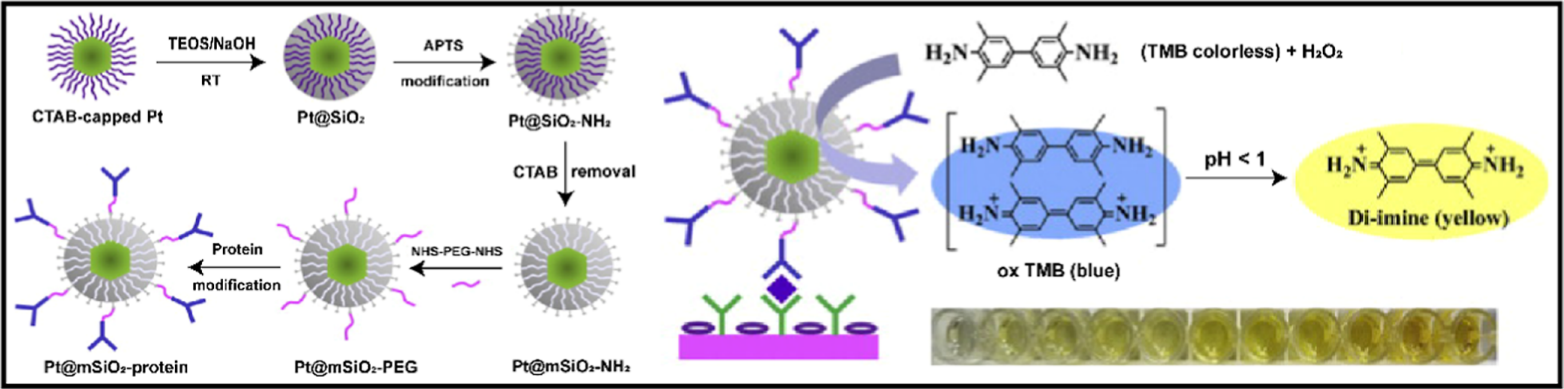
Covalent conjugation in plasmonic ELISA using mesoporous silica-encapsulated platinum nanoparticles for enhanced peroxidase-like activity in colorimetric immunoassays. Reproduced with permission.^109^ Copyright 2015, Elsevier.

Covalent immobilization ensures stable, oriented antibody attachment that significantly enhances sensitivity and reproducibility, making it the preferred method for clinical and commercial plasmonic ELISA platforms.

### 4.3 Affinity-Based Specific Interactions

Affinity-based functionalization utilizes high-affinity biomolecular pairs, primarily the biotin–avidin system, to achieve specific and multivalent antibody attachment. The most common method for affinity-based immobilization is the biotin–avidin interaction (BA).^99,111^ Before the introduction of NLISA, inert NPs (lacking catalytic activity) were commonly used as carriers for antibodies and enzymes to enhance the performance of traditional ELISA.^112–114^ For instance, biotin-labeled IgG was modified onto the surface of AuNP (AuNP@IgG-bio), providing more binding sites for enzymes, thus achieving signal amplification greater than that of a single enzyme. Upon incubation with ALP-labeled streptavidin, more enzyme-labeled antibodies participated in the signal amplification. Successful detection of chloroacetamide herbicides was demonstrated using this immunosensor.^115^ For acetochlor (ATC) detection, after ATC anchoring, ATC antibody (McAb), AuNP@IgG-bio, and ALP-labeled streptavidin were introduced to bind with anchored ATC (Figure 8). Subsequently, AA2P, the OPD, Au nanostar (AuNS), and Ag^+^ were added. Ag deposition on the AuNS resulted in a growth-based p-ELISA with signal read-out via a portable smartphone. Meanwhile, the formation of a fluorescent product between DHA and OPD provided a fluorescent read-out via a spectrofluorometer. Taking advantage of the biotin–SA affinity interaction, Gao et al. developed a highly sensitive NLISA using Pd@Pt core@shell nanodendrites (Pd@Pt NDs) as the nanozyme. SA was covalently conjugated to the surface of Pd@Pt NDs after EDC/NHS activation and was later used to bind biotin-modified anti-IL-6 DAb.^116^ For detecting IL-6, the fabricated ELISA exhibited a dynamic range of 0.05–2 pg/mL with an ultralow detection limit of 0.044 pg/mL (1.7 fM), 21-fold lower than that of conventional HRP-based ELISA. Overall, introducing a biotin–avidin system into nanozyme immunodetection is promising.

**Figure 8:**
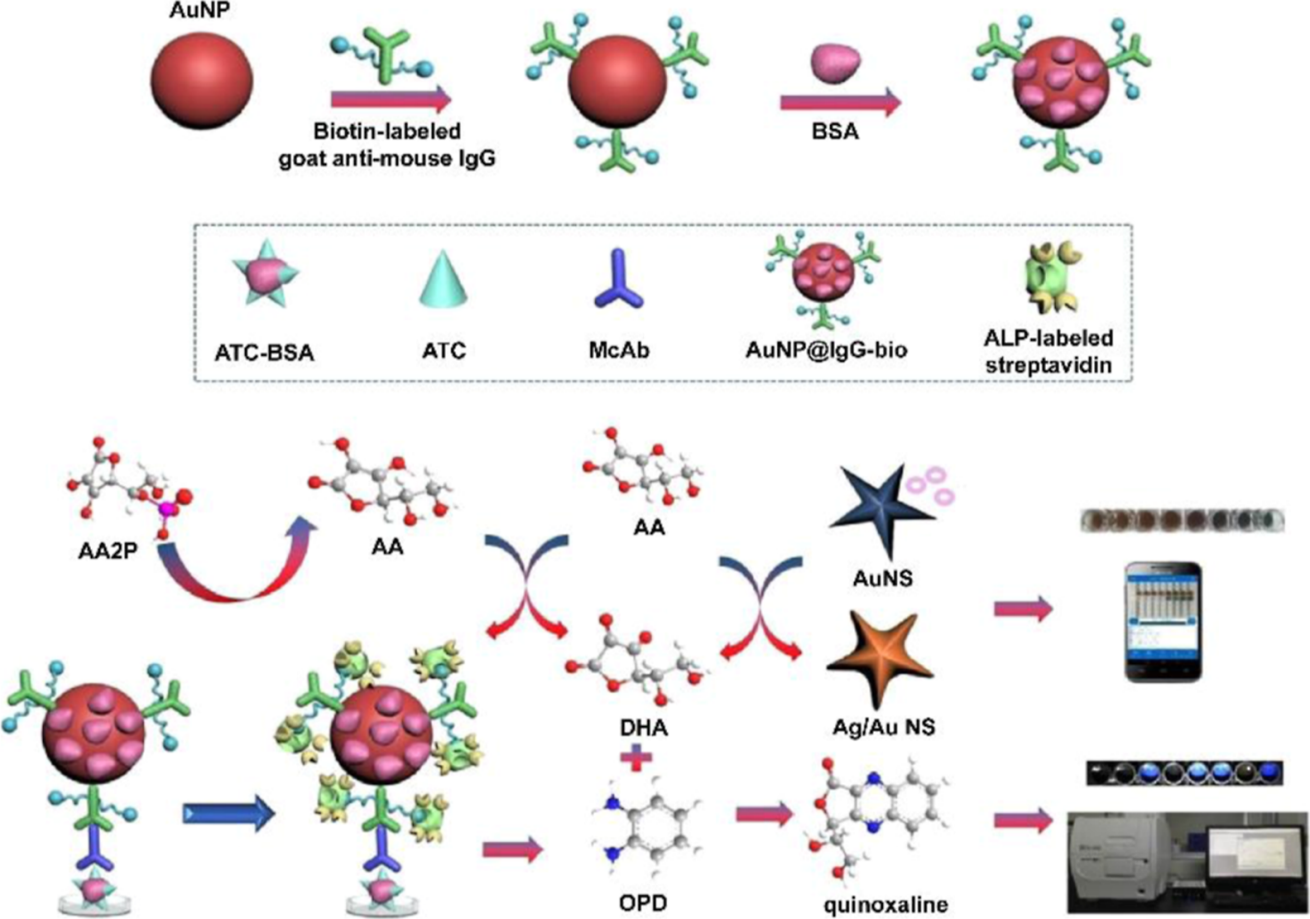
Affinity-based specific interactions in plasmonic ELISA utilizing AuNP@IgG-bio probes for the dual-modal detection of ATC through colorimetric and fluorescent sensing mechanisms. Reproduced with permission.^115^ Copyright 2021, American Chemical Society.

The biotin–avidin system provides a highly specific and amplified means for antibody immobilization, significantly enhancing assay sensitivity and permitting ultralow detection limits; however, it requires meticulous blocking and reagent optimization.

### 4.4 Advanced 2D Materials in p-ELISA

The use of advanced 2D materials, such as graphene and MXenes, in plasmonic ELISA platforms represents a significant advancement in biosensor technology. These materials offer exceptional electrical conductivity, high surface area, and versatile surface chemistries, enabling efficient and stable immobilization of antibodies and biomolecules.^100,101^ Their unique physicochemical properties improve signal transduction and reduce background noise, resulting in enhanced sensitivity and specificity compared with traditional nanoparticle-based systems. Additionally, the mechanical flexibility and scalability of these 2D materials allow for miniaturized, wearable, and point-of-care diagnostic devices, representing a significant step forward in plasmonic immunoassays.

#### 4.4.1 GO and rGO

GO and rGO exhibit exceptional electronic conductivity, an extensive specific surface area, and a rich array of oxygen-containing functional groups that facilitate the immobilization of antibodies through both covalent and noncovalent mechanisms.^117,118^ These attributes enhance the density and stability of biomolecule attachment, which is critical for improving the sensitivity of plasmonic ELISA. Furthermore, graphene-based field-effect transistor immunosensors have demonstrated the capability for rapid, ultrasensitive detection of allergens and biomarkers at picogram levels.^33^ Composite materials that integrate GO/rGO with conducting polymers or metal sulfides further augment electron transfer and minimize background interference, thereby enabling label-free detection platforms that are compatible with miniaturized, point-of-care diagnostics.^117,118^ The biocompatibility and flexibility of graphene derivatives render them outstanding candidates for wearable biosensors and integrated plasmonic ELISA devices. Additionally, graphene derivatives provide superior surface functionalization and electronic properties, significantly advancing plasmonic ELISA biosensors’ sensitivity, selectivity, and versatility, with promising applications in rapid and wearable diagnostics.^33,101,117–119^

#### 4.4.2 MXenes

MXenes, a family of 2D transition metal carbides and nitrides, exhibit metallic conductivity, hydrophilicity, and versatile surface chemistries, enabling efficient antibody immobilization and rapid electron transfer.^120^ These properties translate into improved electrochemical immunosensors with low detection limits and broad linear ranges for clinically relevant biomarkers such as carcinoembryonic antigen (CEA) and interleukin-6 (IL-6).^32,100^ Hybrid composites combining MXenes with multiwalled carbon nanotubes or gold nanoparticles enhance active surface area and electron transfer, mitigating MXene aggregation and improving biosensor stability.^100^ Functionalization strategies incorporating electroactive tags like Prussian blue and gold nanostructures enable robust, label-free electrochemical ELISA platforms with superior sensitivity, selectivity, and reproducibility suitable for clinical and point-of-care settings.^32^ MXenes represent a cutting-edge class of materials for plasmonic ELISA functionalization, combining outstanding electronic and surface properties to create ultrasensitive, stable, and rapid biosensors poised for clinical translation.

To provide a clear and comprehensive comparison of the various surface functionalization methodologies and types of plasmonic nanostructures discussed previously, Table 2 summarizes their mechanisms, primary advantages, limitations, and reported performance metrics in plasmonic ELISA applications. This integrated overview highlights how chemical functionalization techniques and nanoparticle design together influence assay sensitivity, specificity, stability, and practical usability. Understanding these factors in juxtaposition enables informed decisions in the development of biosensors tailored to specific diagnostic goals.

**Table 2:** Comparative Performance of Surface Functionalization Strategies for Plasmonic Nanomaterials in ELISA

functionalization method	mechanism	advantages	limitations	performance highlights	comparative note	ref.
non-covalent adsorption	electrostatic, hydrophobic, van der Waals, H-bonding	simple, mild preserves nanozyme activity	uncontrolled orientation, nonspecific adsorption	LOD improvement × 1000 (e.g., measles IgM), moderate assay stability	baseline sensitivity, and reproducibility	^105^
covalent conjugation	Au–S bonds, EDC/NHS carbodiimide chemistry	strong, stable attachment, oriented binding	requires optimized chemistry, risk of denaturation	140-fold sensitivity increase, improved reproducibility and stability	high stability, preferred for clinical-grade assays	^109^
						^110^
affinity-based interactions	biotin–avidin (streptavidin) system	highly specific, multivalent binding, amplification	steric hindrance, reagent cost, complex blocking	Ultralow LOD (0.044 pg/mL for IL-6), strong signal amplification	powerful amplification, complexity, and cost are higher	^115^
						^116^
graphene derivatives (GO/rGO)	covalent/noncovalent on conductive 2D sheets	high surface area, excellent conductivity, flexible	potential nonspecific binding, requires passivation	Picogram LODs; rapid response, label-free sensing, flexible formats	significant sensitivity and speed improvements, enables miniaturization	^117,118^
MXenes	metallic conductivity, hydrophilic 2D surfaces	ultrahigh conductivity, biocompatible, tunable chemistry	aggregation tendency, synthesis complexity	Sub-pg/mL detection limits, wide range, rapid, label-free electrochemical sensing, high stability	superior electrochemical properties, emerging materials with high promise but synthesis challenges	^32,100^

As indicated in Table 2, traditional methodologies, such as noncovalent and covalent functionalization, continue to serve as robust platforms for the immobilization of antibodies, effectively balancing ease of use and reproducibility. Affinity-based systems additionally enhance signals through highly specific interactions. Furthermore, emerging 2D materials, including graphene derivatives and MXenes, markedly improve sensitivity and facilitate miniaturized, rapid, and label-free detection formats; however, challenges such as aggregation and surface passivation persist. Consequently, the selection of an optimal functionalization and nanoparticle strategy necessitates careful consideration of assay complexity, performance requirements, and scalability, thereby guiding the design of next-generation plasmonic ELISA systems with enhanced clinical and point-of-care applicability.

## 5 New Modalities Endowed by Plasmonic Nanostructures

Plasmonic nanomaterials like gold and silver nanoparticles enhance conventional ELISA by introducing diverse transduction mechanisms that utilize their unique optical and electronic properties.^6^ A key aspect of these advancements is LSPR, where the collective oscillations of conduction electrons focus electromagnetic energy into nanoscale “hot spots”.^7^ This effect significantly boosts signals such as fluorescence and Raman scattering, facilitates efficient photothermal conversion, and improves electrochemical interfaces.^8,9^ By leveraging these mechanisms, five advanced ELISA modalities have emerged: electrochemical, microfluidic, photothermal, SEIRA, and SERS. Moreover, innovations have integrated CRISPR-Cas12a molecular amplification with plasmonic readouts for wearable and point-of-care (POC) formats, thereby redefining the sensitivity, specificity, speed, and multiplexing capabilities in immunoassays.

### 5.1 Electrochemical ELISA (E-ELISA)

Electrochemical ELISA (E-ELISA) represents an innovative technique for the sensitive and selective detection of biomarkers, which is particularly advantageous in liquid biopsy applications. This method integrates the specificity of traditional ELISA with the heightened sensitivity and simplicity afforded by electrochemical detection.^121^ In contrast to conventional ELISA, which depends on optical signals, E-ELISA identifies electroactive products generated by enzyme-mediated reactions directly at the electrode surface. Typically, capture antibodies are immobilized on conductive electrodes, such as gold, carbon, or graphene. Upon binding of the target biomarker to the capture antibody, a secondary antibody conjugated with an enzyme is introduced to create a sandwich complex. The enzyme catalyzes the generation of an electroactive species, which is subsequently detected utilizing voltammetric or amperometric techniques, yielding a quantitative electrochemical signal.^122,123^

For instance, Wang et al. developed an electrochemical immunosensor aimed at detecting interleukin-6 (IL-6), a biomarker correlated with subarachnoid hemorrhage (SAH). As illustrated in Figure 9, AuNPs modified with thiol (THI) groups were electrodeposited onto a glassy carbon electrode, followed by the immobilization of anti-IL-6 antibodies and subsequent blocking with bovine serum albumin (BSA). The binding of IL-6 resulted in a concentration-dependent reduction in current due to enhanced electron transfer blocking. The sensor exhibited a broad detection range spanning from 0.01 to 100 ng/mL, with a low LOD calculated at 1.85 pg/mL.^124^ Similarly, peptide-based electrochemical sensors and gold nanostructured electrodes have been developed for biomarkers associated with rheumatoid arthritis and blood cancer, respectively, demonstrating superior sensitivity and reproducibility compared to commercial ELISA kits.^125,126^

**Figure 9:**
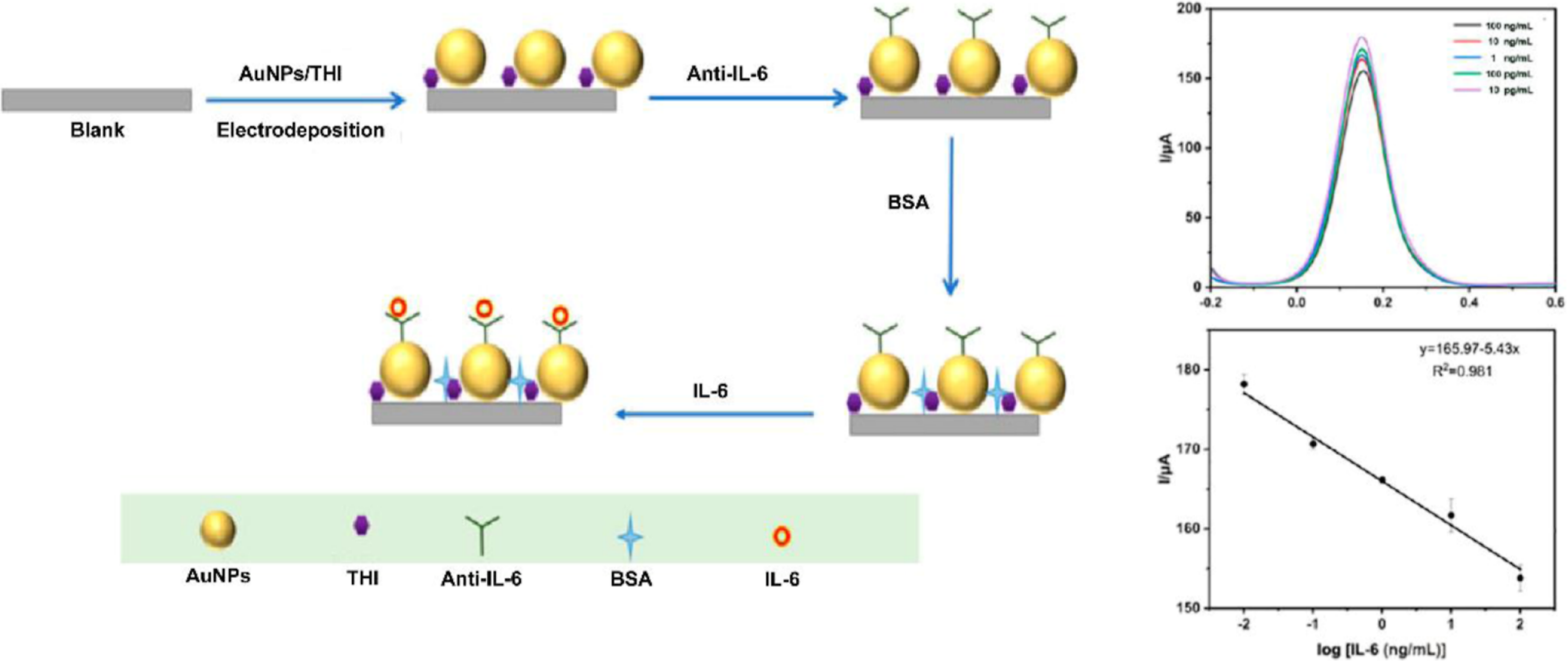
Electrochemical ELISA for IL-6 detection using thiol-modified AuNPs and anti-IL-6 antibodies. AuNPs are electrodeposited on a glassy carbon electrode, followed by antibody immobilization and BSA blocking. IL-6 binding results in a concentration-dependent decrease in electrochemical current, as shown in the current (*I*) vs potential (*E*) curves. The calibration plot shows a linear detection range from 10 pg/mL to 100 ng/mL, indicating high sensitivity and specificity. Reproduced with permission.^124^ Copyright 2023, Frontiers.

Nanostructured electrodes that are modified with plasmonic nanoparticles, such as AuNPs, significantly enhance electron transfer kinetics and increase the availability of antibody binding sites, thereby facilitating detection limits in the picogram per milliliter range. The integration of microfluidics with flexible substrates in electrochemical enzyme-linked immunosorbent assay (E-ELISA) further supports the miniaturization and portability of assays without compromising their performance.^122,123^ These advancements establish electrochemical ELISA as a promising platform for rapid, sensitive, and multiplexed clinical diagnostics.

### 5.2 Microfluidic ELISA

Microfluidic ELISA effectively miniaturizes traditional immunoassays by facilitating antigen–antibody binding and detection within microchannels or microwells at nanoliter to picolitre volumes.^127^ This method offers sensitivity that is 100 to 1000 times greater than that of conventional ELISA techniques, which typically utilize sample volumes ranging from 50 to 200 μL.^128^ The assay workflow closely resembles that of traditional ELISA: capture antibodies are immobilized on the surfaces of the microchannels; nonspecific binding sites are blocked, and samples containing target antigens flow through the device, leading to the formation of antigen–antibody complexes. Detection antibodies, labeled with enzymes or fluorophores, bind to these complexes, generating measurable signals, whether colorimetric, fluorescent, or enzymatic, thereby enabling quantitative analysis.^128–130^

For instance, Yang et al. developed a multiplex microfluidic paper-based device (multi-μPAD) that is capable of quantifying allergen-specific immunoglobulin E (sIgE) in serum, with an LOD of 0.246 KUA/L (Figure 10).^131^ This platform has demonstrated superior accuracy compared with commercial ELISA kits when evaluated with samples from allergy patients. In a separate study, Gu et al. integrated microfluidics with SERS detection to sensitively quantify cervical cancer biomarkers, specifically SCCA and carcinoembryonic antigen (CEA), down to subpicogram per milliliter levels in serum.^132^ Furthermore, Zhang et al. illustrated a microfluidic ELISA that employs bifunctional gold@Prussian blue nanoparticles for the rapid and automated detection of the milk allergen alpha-lactalbumin (α-LA), achieving a detection limit of 0.011 ng/mL through the use of dual colorimetric and SERS signals.^133^ These studies collectively underscore the potential of microfluidic ELISA technology to facilitate rapid, sensitive, multiplexed, and portable immunoassays, making them well-suited for point-of-care diagnostics.

**Figure 10:**
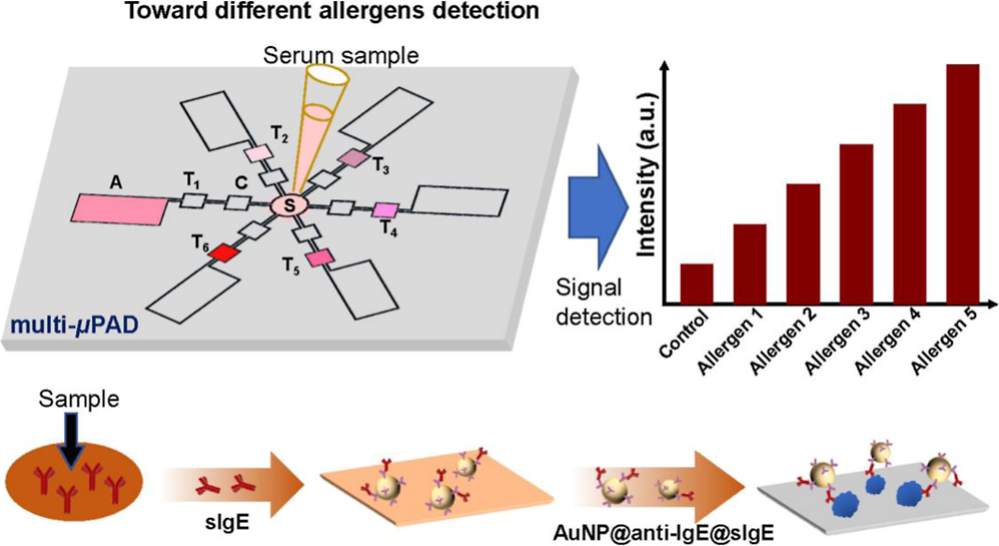
Microfluidic enzyme-linked immunosorbent assay (ELISA) on a paper-based device enables multiplexed colorimetric detection of allergens. Serum samples enter the sample pad (S) and pass through the conjugate (C), test (T), and absorbent (A) pads. AuNPs with anti-IgE antibodies (AuNP@anti-IgE) on the conjugate pad bind to allergen-specific immunoglobulin E (IgE) in the sample. Various allergens on the test pads capture these complexes, resulting in color changes that are proportional to the concentration of the allergens. Reproduced with permission.^131^ Copyright 2023, Elsevier.

### 5.3 Photothermal ELISA

Photothermal ELISA represents a sophisticated immunoassay technique that capitalizes on the photothermal effect, whereby plasmonic nanomaterials transform absorbed light into localized thermal energy. The consequent increase in temperature functions as the detection signal, which correlates directly with the concentration of target biomolecules. Photothermal agents, including Prussian blue nanoparticles and gold nanorods, are conjugated to antibodies. Upon binding to target antigens, these agents are irradiated by near-infrared (NIR) light. The heat generated results in an increase in the solution’s temperature, which is meticulously measured by a thermometer or infrared sensor, thereby yielding a highly sensitive and interference-resistant readout.^134,135^

For instance, Wang et al. developed a photothermal ELISA for the detection of ochratoxin A (OTA) utilizing AA-mediated in situ growth of gold nanostars (AuNSs). As depicted in Figure 11, in the absence of OTA, monoclonal antibodies (mAb) adhere to BSA-OTA immobilized on the microplate, facilitating the capture of secondary antibody-labeled HRP (sAb-HRP). The HRP catalyzes the production of AA, which subsequently reduces Au­(III) ions to Au(0) atoms on the gold seeds, leading to the formation of AuNSs in conjunction with Ag^+^ ions. Upon near-infrared (NIR) laser irradiation, these AuNSs generate a photothermal signal by increasing the temperature in direct proportion to the target concentration. Conversely, in the presence of OTA, competitive binding diminishes mAb attachment, resulting in a decrease in AuNS formation and a subsequent decrease in temperature elevation. This assay attained an ultrasensitive detection limit of 28 fg/mL for OTA, demonstrating excellent recovery in spiked food samples.^136^ Similarly, Sheng et al. developed a dual-mode immunochromatographic assay utilizing MoS_2_@Au nanocomposites to detect imidacloprid (IMI) in food products. This assay integrates colorimetric and photothermal signals by employing antibody-modified MoS_2_@Au probes stimulated by 808 nm laser excitation. The detection limits are 12 μg/L for visual and 0.18 μg/L for photothermal detection, facilitating sensitive and rapid quantification. The method relies on competitive binding between IMI and the immobilized antigen present on the test strip.^137^ These studies demonstrate that photothermal ELISA offers rapid, highly sensitive, and reliable quantification of low-abundance biomarkers, providing significant advantages in complex sample matrices due to minimal optical background interference.

**Figure 11:**
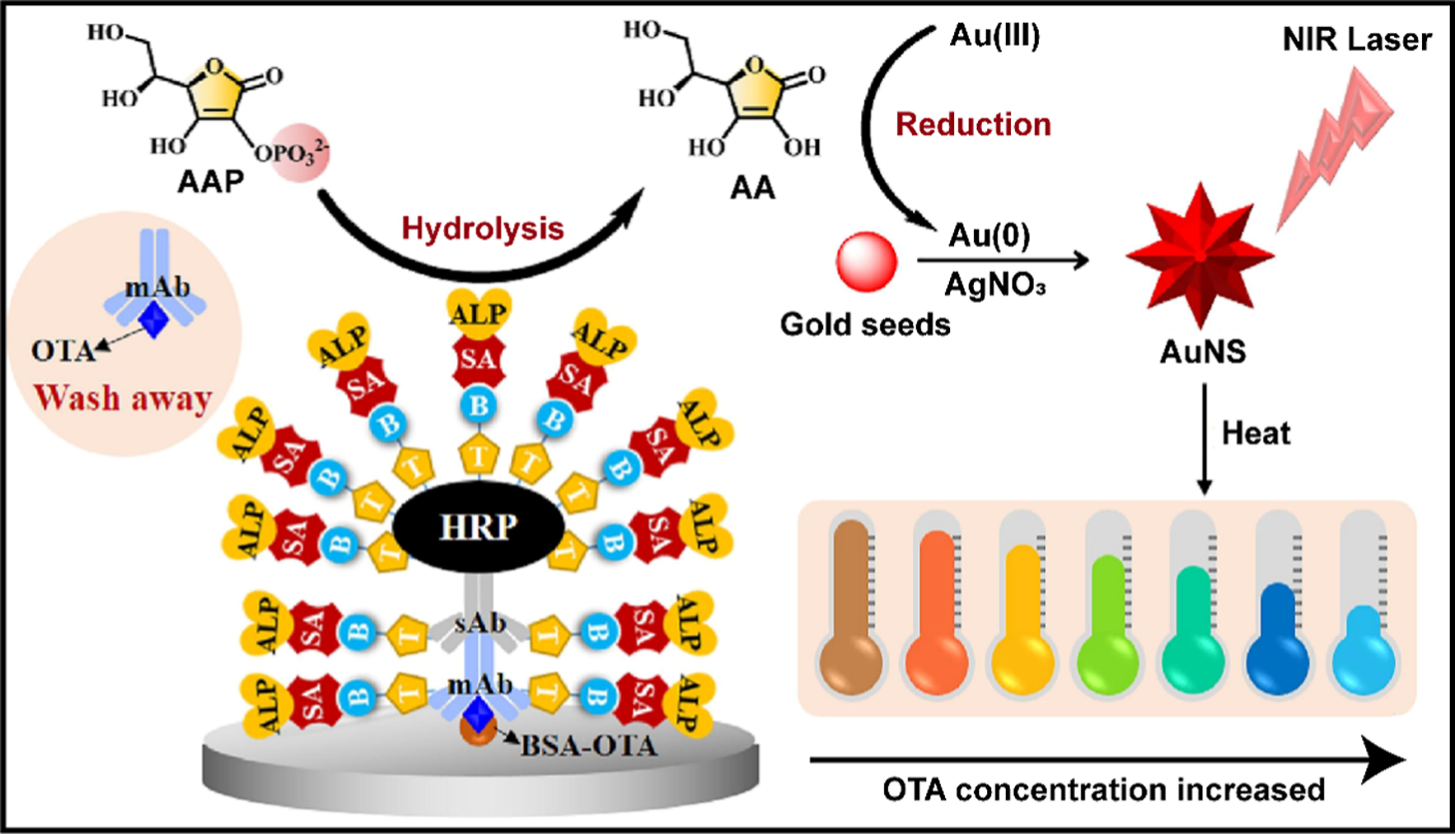
Schematic illustrating the photothermal Enzyme-Linked Immunosorbent Assay (ELISA) for the detection of ochratoxin A (OTA). HRP catalyzes the formation of AA, which subsequently reduces Au­(III) ions to Au(0) on gold seeds, facilitating the generation of gold nanostars (AuNSs). Near-infrared (NIR) laser irradiation induces AuNSs to emit a photothermal signal, which is manifested by an increase in solution temperature. OTA acts as an inhibitor of antibody binding, resulting in a decrease in AuNS formation and a reduction in temperature rise, thus enabling the ultrasensitive detection of OTA. Reproduced with permission.^136^ Copyright 2023, Elsevier.

### 5.4 SEIRA-ELISA: Surface-Enhanced Infrared Absorption Immunoassay

SEIRA-ELISA employs surface-enhanced infrared absorption (SEIRA) to amplify infrared-active vibrational signals for ultrasensitive immunoassay detection.^138,139^ In contrast to conventional ELISA performed on microplates, SEIRA-ELISA immobilizes target antibodies on plasmonic nanostructured substrates, which enhance the vibrational absorption of enzymatically generated products or biomolecules. The strong coupling between molecular vibrations and plasmonic nanostructures enhances the light–matter interaction, significantly improving the infrared absorbance signals. This amplification facilitates antigens’ sensitive identification and quantification by detecting their unique vibrational “fingerprint” spectra.^138–140^

For instance, Bao et al. developed an attenuated total reflection (ATR)-SEIRA platform by depositing AuNPs onto a zinc selenide (ZnSe) prism to monitor DNA hybridization in real time.^141^ As illustrated in Figure 12, this Au/ZnSe substrate facilitated enhanced infrared absorption across a broad spectral range, encompassing the fingerprint region distinctive for molecular identification. The platform achieved a DNA hybridization rate constant of 1.52 ± 0.06 × 10^4^ M^–1^ s^–1^ and an antibody–antigen association rate constant of 4.94 × 10^5^ M^–1^ s^–1^, thereby demonstrating both sensitivity and kinetic resolution. Furthermore, Yilmaz et al. utilized SEIRA-ELISA employing colloidal silver nanoparticles (AgNPs) to differentiate microbial species such as , , and by their unique vibrational spectra, underscoring SEIRA’s potential in pathogen identification.^142^

**Figure 12:**
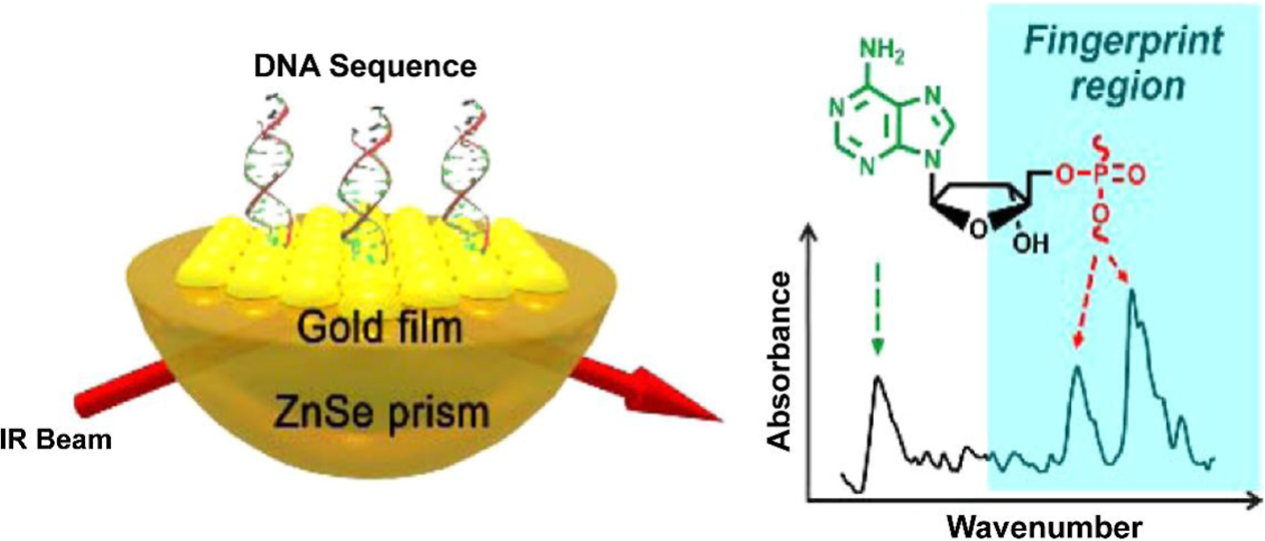
A Schematic representation of SEIRA-ELISA provided for the detection of DNA hybridization, utilizing a gold (Au) nanoparticle film deposited on a zinc selenide (ZnSe) prism within the framework of the attenuated total reflection surface-enhanced infrared absorption spectroscopy (ATR-SEIRAS) platform. The employment of the plasmonic substrate enhances infrared absorption, thereby facilitating sensitive spectral monitoring of DNA hybridization within the fingerprint region. Reproduced with permission.^141^ Copyright 2018, American Chemical Society.

### 5.5 SERS-ELISA: Surface-Enhanced Raman Scattering Immunoassay

SERS-ELISA employs SERS to detect Raman-active vibrational signals that are significantly amplified in proximity to plasmonic nanostructures.^13^ In this method, plasmonic nanoparticles are modified with Raman reporter molecules, thereby functioning as SERS tags. This method provides highly sensitive and specific detection of low-abundance biomolecules, often exceeding the sensitivity of traditional ELISA assays. The considerable electromagnetic enhancement and chemical enhancement effects present in nanogaps and on sharp nanostructures facilitate detection limits at the single-molecule level under optimal conditions.^104,143^

For instance, Yu et al. developed a surface-enhanced Raman spectroscopy enzyme-linked immunosorbent assay (SERS-ELISA) platform for the detection of the SARS-CoV-2 nucleocapsid protein (N-protein). As depicted in Figure 13, silica-encapsulated gold core–satellite nanoparticles (CS@SiO_2_) conjugated with detection antibodies bind to the N-protein captured in either 96-well or 384-well plates. The intensity of the Raman signal emitted by the reporter molecule (1,4-benzenedimethanethiol) is directly proportional to the concentration of the N-protein. Among the various SERS nanotags evaluated, CS@SiO_2_ exhibited the most robust signal and reached an LOD of 8.81 plaque-forming units per milliliter (PFU/mL), which is approximately ten times more sensitive than traditional ELISA.^144^ In a separate investigation, Zhai et al. introduced an innovative SERS-ELISA (ELI-SERS) sensor that combines ELISA with SERS, designed for detecting myoglobin (Myo) in serum. The detection mechanism produces a sandwich complex with Myo binding to capture antibodies on the plate and to gold nanoparticles tagged with a Raman dye (IR-808), thus amplifying the Raman signal. This system allows for rapid and highly sensitive detection in just 6 min, eliminating the need for complex sample processing. The sensor has a detection limit as low as 5.3 pg/mL and demonstrates exceptional specificity and accuracy for clinical applications.^145^ Similarly, Zhai et al. conducted a comparison of various AuNPs shapes for cortisol detection employing magnetically assisted SERS immunoassays.^145^ Lee et al. developed a SERS-ELISA platform using plasmonic Au nanotrenches with ∼1 nm gaps to enhance the Raman signal sensitivity for SARS-CoV-2. This method achieves a detection limit of 1 fg/mL, outpacing traditional ELISA by 6 orders of magnitude. It utilizes specific antibody–antigen interactions to generate strong, selective Raman signals for quick and accurate virus identification.^146^

**Figure 13:**
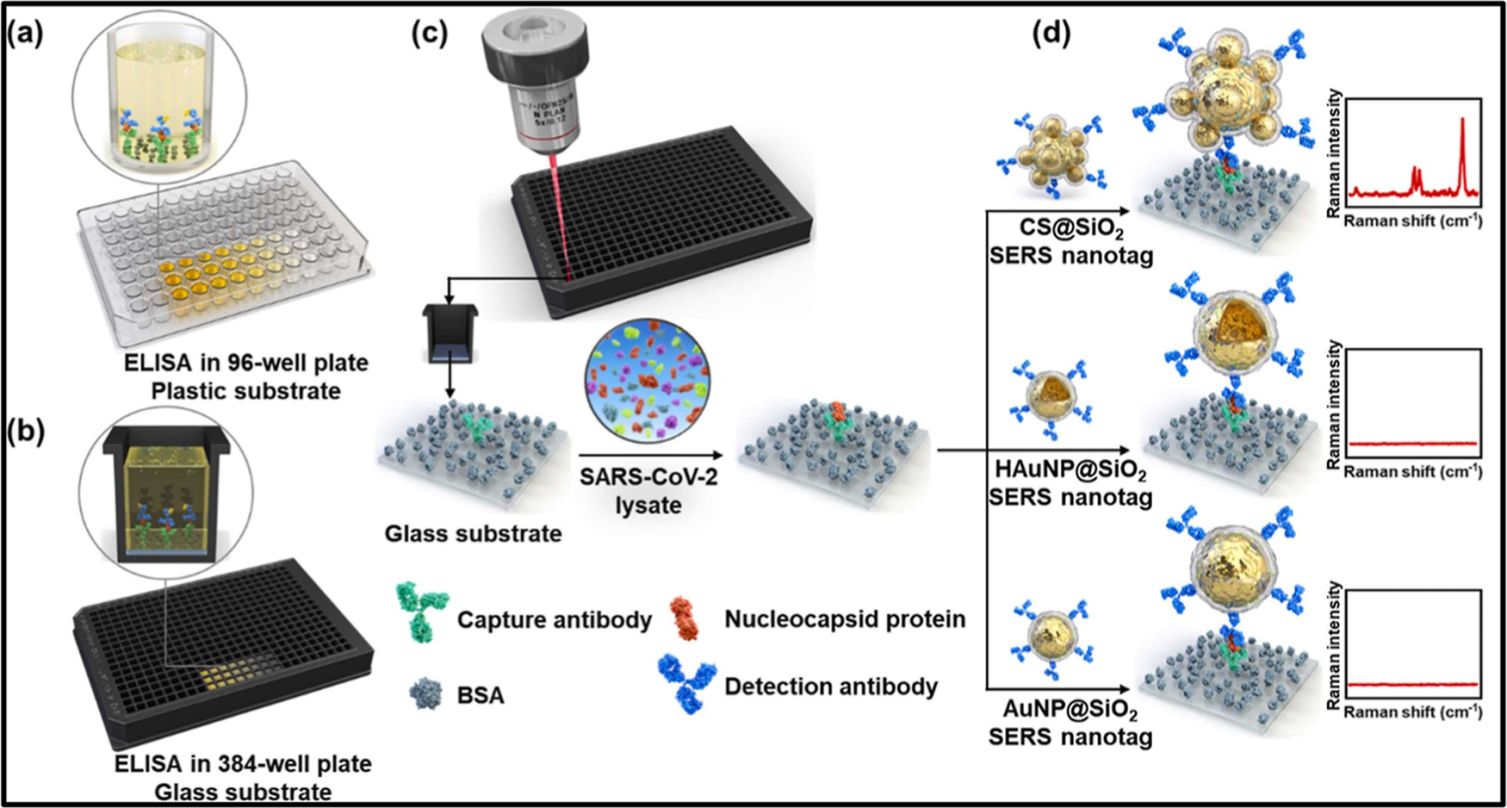
Schematic of SERS-ELISA for the detection of the SARS-CoV-2 nucleocapsid protein. (a) Conventional ELISA was performed on a 96-well plastic plate. (b) ELISA was conducted on a 384-well glass plate for increased throughput. (c) SERS-ELISA setup featuring SERS nanotags on a 384-well plate, enhancing detection sensitivity. (d) Comparative evaluation of SERS nanotags CS@SiO_2_, HAuNP@SiO_2_, and AuNP@SiO_2_, demonstrating a superior signal from CS@SiO_2_ in their Raman spectra. Reproduced with permission.^144^ Copyright 2023, Elsevier.

### 5.6 CRISPR-Enabled Plasmonic ELISA

The programmable trans-cleavage activity of CRISPR-Cas12a has been innovatively integrated with plasmonic ELISA platforms, resulting in assays that combine the specificity of molecular recognition with nanoplasmonic signal amplification for the ultrasensitive quantification of biomarkers.^147^ The core mechanism involves target binding that activates Cas12a’s nonspecific single-stranded DNA (ssDNA) cleavage function. This trans-cleavage operates on multiple ssDNA reporters tethered to plasmonic nanoparticles, such as gold nanostars, modifying plasmonic signals, including fluorescence and SERS intensities, which facilitates signal amplification that exceeds traditional ELISA capabilities.^35^ In the S-CasLISA platform, antibodies are responsible for capturing protein targets, while DNA-tagged detection antibodies instigate the activation of Cas12a. Cas12a subsequently cleaves the SERS reporter DNA on gold nanostars, permitting the detection of PSA down to 0.17 pg/mL, yielding an approximately 1400-fold improvement over conventional ELISA methods. Moreover, programmable DNA nanoswitches have been developed to facilitate the multiplexed detection of both nucleic acid and protein biomarkers using a single crRNA, resulting in nearly 100-fold sensitivity enhancements.^148^ Additionally, MXene–gold dual-mode sensors integrated with the CRISPR/Cas12a system that incorporate fluorescence quenching and SERS enhancement have demonstrated rapid endotoxin detection at 15.9 pg/mL within a time frame of 30 min.^149^ Despite challenges such as the complexity of reagents and the temperature sensitivity of Cas enzymes, advancements in freeze-dried reagents and ambient-temperature Cas variants are expediting clinical translation. These CRISPR-integrated plasmonic ELISA systems signify a promising Frontier in multiplexed, rapid, and highly sensitive biomarker detection, making them suitable for point-of-care diagnostics and personalized medicine.

### 5.7 Wearable and Point-of-Care Sensor Platforms

Integrating plasmonic ELISA chemistry into portable, cost-effective formats is crucial for real-world clinical impact. Wearable biosensors equipped with microfluidic channels can continuously collect biofluids such as sweat and direct them across plasmonic sensors functionalized with antibodies or aptamers. When biomarkers bind (e.g., cortisol, glucose, cytokines), they induce shifts in LSPR or generate enhanced Raman scattering signals, which can be detected using compact spectrometers or smartphone cameras, allowing for noninvasive, real-time monitoring.^150^ Paper-based lateral flow assays enhanced with plasmonic nanoparticles replace traditional gold lines with functionalized probes, improving signal visibility and enabling quantitative analysis through simple optical scanners. These point-of-care (POC) platforms deliver rapid results in about 15 min without bulky instrumentation, making them ideal for decentralized diagnostics in resource-limited areas.^37^ Recent advancements include flexible, multiplexed electrochemical biosensors modified with GO and gold nanoparticles, which achieve high sensitivity for inflammatory biomarkers such as IL-6 and TNF-α.^122^ Wearable nanozyme–enzyme electrochemical biosensors on flexible substrates such as polyimide films enable continuous lactate monitoring during exercise, validated against standard ELISA methods.^39^ Innovative microfluidic SERS patches made from biocompatible silk fibroin facilitate noninvasive sweat cortisol monitoring with high sensitivity and stability using aptamer probes to enhance specificity. These platforms showcase the integration of plasmonic sensing with flexible materials and microfluidics, broadening the potential for personalized health monitoring and stress assessment.^150^

These wearable POC sensor platforms exemplify the merging of plasmonic nanotechnology, flexible electronics, and microfluidics to create rapid, sensitive, and user-friendly diagnostic devices. Future research will emphasize enhancing multiplexing, durability, and real-world validation to advance plasmonic ELISA toward routine clinical and home healthcare applications.

## 6 Clinical Trials and Real-World Case Studies

Recent years have witnessed substantial advancements in the validation of plasmonic-nanomaterial-enabled biosensors within clinical environments, thereby illustrating their practical utility in facilitating early diagnosis, ongoing disease monitoring, and personalized medicine. These developments have proven to be particularly significant in detecting crucial biomarkers associated with cancer, infectious diseases, and cardiovascular conditions.

### 6.1 Cancer Biomarkers

Plasmonic biosensors that are designed for the detection of cancer biomarkers, including PSA, carcinoembryonic antigen (CEA), and GFAP, have demonstrated exceptional sensitivity and specificity in clinical trials.^111^ Aptamer-functionalized SERS sensors that utilize gold/silicon (Au/Si) nanoarrays have achieved femtomolar detection of gastric cancer markers within 18 min, which has been validated against the standard ELISA in patient serum samples.^151^ Electrochemical immunosensors that utilize MXene, carbon nanotubes, and gold nanoparticle composites have consistently quantified CEA down to 0.015 ng/mL in clinical serum samples, exhibiting excellent correlation (*R* ^2^ > 0.98) with traditional assays.^100^ Additionally, multiplexed detection platforms that employ DNA origami plasmonic nanoantennas have facilitated the simultaneous monitoring of immunotherapy-related cytokines, such as tumor necrosis factor-alpha (TNF-α) and interferon-gamma (IFN-γ), in biological fluids, thereby providing real-time insights into tumor microenvironments.^152^ Collectively, these studies substantiate the clinical translational potential of plasmonic biosensors for early cancer detection and therapeutic monitoring.

### 6.2 Infectious Disease Markers

The COVID-19 pandemic has accelerated the clinical deployment of plasmonic biosensors for the detection of viral antigens and antibodies. LSPR nanosensors functionalized with gold nanorods have detected SARS-CoV-2 antibodies with sensitivity and specificity exceeding 95% in patient cohorts, thereby enabling the identification of early stage infections within 10 days of symptom onset.^153^ Hybrid plasmonic-fluorescence platforms that combine nucleic acid amplification with immunoassays have simultaneously detected hepatitis B, monkeypox, and HIV, achieving clinical sensitivity and specificity levels over 98%, validated on more than 200 patient samples.^154^ Furthermore, plasmonic nanoceria composites have facilitated rapid, on-site colorimetric detection of Ebola glycoprotein at picomolar concentrations, demonstrating a concordance rate of 95% with PCR tests during field trials.^155^ Additionally, plasmonic ELISA detects infectious disease markers using plasmonic metasurfaces, such as gold nanopyramids, to enhance signals via LSPR. This method enables the ultrasensitive identification of biomarkers, such as the dengue NS1 protein and tuberculosis antigens, achieving detection limits ranging from picomolar to femtomolar levels. It greatly surpasses the sensitivity of traditional ELISA, facilitating swift and precise diagnosis.^106^ These real-world validations underscore the role of plasmonic biosensing as a powerful tool for multiplexed and rapid diagnostics of infectious diseases applicable in both centralized laboratories and resource-limited settings.

### 6.3 Cardiac Markers

Cardiac troponin I (cTnI) remains the gold standard biomarker for diagnosing myocardial infarction and for timely clinical decision-making. Plasmonic biosensors have advanced the detection of cTnI by integrating nanomaterials that enhance signal transduction and assay speed.^45^ Electrochemical sensors incorporating nickel vanadate and rGO composites have achieved detection limits as low as 2 pg/mL in clinical plasma samples, matching or exceeding the high sensitivity of ELISA benchmarks.^156^ Microfluidic plasmonic chips that employ gold nanobipyramids integrate thermoplasmonic effects with LSPR and SERS to deliver sensitive detection within 5 min. These platforms have demonstrated 100% specificity and approximately 75% sensitivity in over 50 clinical samples, comparable to conventional assays.^157^ Additionally, SERS-based microfluidic immunoassays have further enhanced sensitivity and multiplexing capabilities by simultaneously detecting cTnI and cardiac myoglobin, offering rapid, portable, and ultrasensitive detection that is essential for acute cardiac care.^158^

The findings obtained from these clinical trials and real-world case studies collectively substantiate that biosensors empowered by plasmonic nanomaterials facilitate the rapid, ultrasensitive, and multiplexed detection of disease biomarkers with clinical precision. Their demonstrated efficacy in patient samples, coupled with their compatibility with point-of-care formats, positions them as transformative instruments capable of enhancing early diagnosis, disease monitoring, and personalized treatment across various medical fields. Ongoing advancements in scalable manufacturing, device integration, and regulatory approval processes will be imperative for their widespread clinical adoption and impact.

## 7 Conclusion

Plasmonic enzyme-linked immunosorbent assay (p-ELISA) has emerged as a versatile and powerful platform that integrates noble metal nanostructures with conventional immunoassays to facilitate rapid, ultrasensitive, and multiplexed biomarker detection. By harnessing the phenomena of LSPR, which is modulated through biocatalytic activity and target binding, plasmonic ELISAs enable signal amplification through mechanisms such as growth, etching, and aggregation, thereby overcoming the sensitivity limitations inherent in traditional ELISA. These methodologies have demonstrated significant improvements in the LOD, ranging from 10-fold to over 1000-fold, with detection thresholds achieving picogram per milliliter (pg/mL) levels. Advanced surface functionalization techniques, including covalent conjugation and affinity-based binding, ensure that antibody immobilization is stable and oriented while preserving the catalytic activity of nanozymes, thus enhancing assay reproducibility and specificity. The integration of emerging two-dimensional nanomaterials, such as graphene and its derivatives and MXenes, has further augmented sensitivity (with LOD improvements of up to 200-fold), signal stability, and facilitated device miniaturization compatible with point-of-care applications. Furthermore, novel assay formats including electrochemical, microfluidic, photothermal, SEIRA, SERS, and CRISPR-enabled ELISAs have broadened detection modalities, multiplexing capabilities, and assay speed, achieving detection times as minimal as a few minutes and allowing for multiplexed analyte quantification with femtomolar sensitivity. Clinical validations and real-world case studies have corroborated the potential of plasmonic ELISAs in the early detection of cancer, infectious disease diagnostics, and cardiovascular biomarker monitoring, demonstrating sensitivity and specificity comparable to or superior to those of traditional gold-standard methods. Nevertheless, challenges persist regarding assay standardization, reproducibility, multiplex integration, and scalable manufacturing. Addressing these challenges through nanomaterial synthesis advancements, surface chemistry optimization, and device engineering will be pivotal for the successful clinical translation of these platforms. This review synthesizes these advancements, emphasizing the transformative potential of plasmonic ELISA. Continued interdisciplinary innovation will propel the development of robust, cost-effective, and user-friendly diagnostic tools capable of revolutionizing personalized medicine and global health monitoring.
